# Diagnostic accuracy of convolutional neural networks in classifying hepatic steatosis from B-mode ultrasound images: a systematic review with meta-analysis and novel validation in a community setting in Telangana, India

**DOI:** 10.1016/j.lansea.2025.100644

**Published:** 2025-07-31

**Authors:** Akshay Jagadeesh, Chanchanok Aramrat, Santosh Rai, Fathima Hana Maqsood, Adarsh Kibballi Madhukeshwar, Santhi Bhogadi, Judith Lieber, Hemant Mahajan, Santosh Kumar Banjara, Alexandra Lewin, Sanjay Kinra, Poppy Mallinson

**Affiliations:** a Department of Non-communicable Disease Epidemiology, Faculty of Epidemiology and Population Health, London School of Hygiene & Tropical Medicine, London, WC1E 7HT, UK; b Department of Radiology, Kasturba Medical College Mangalore, Manipal Academy of Higher Education, Karnataka, 576 104, India; c NMC Specialty Hospital, Al Ain, P.O. Box: 84142, Abu Dhabi, United Arab Emirates; d Yenepoya Medical College, Yenepoya (Deemed to be University), Mangaluru, Karnataka, 575 018, India; e Indian Council of Medical Research—National Institute of Nutrition, Hyderabad, 500007, Telangana, India; f Department of Medical Statistics, Faculty of Epidemiology and Population Health, London School of Hygiene & Tropical Medicine, London, WC1E 7HT, UK

**Keywords:** Artificial intelligence, Machine learning, Convolutional neural networks, Fatty liver disease, Ultrasonography

## Abstract

**Background:**

Ultrasound is a widely available, inexpensive, and non-invasive modality for evaluating hepatic steatosis (HS). However, the scarcity of radiological expertise limits its utility. Convolutional Neural Networks (CNNs) have potential for automated classification of HS using B-mode ultrasound images. We aimed to assess their diagnostic accuracy and generalisability across diverse study settings and populations.

**Methods:**

We systematically reviewed two biomedical databases up to Dec 12, 2023, to identify studies that applied CNNs in the classification of HS using B-mode ultrasound images as input (PROSPERO: CRD42024501483). We supplemented this review with a novel analysis of the community-based Andhra Pradesh Children and Parents’ Study (APCAPS) in India to address the overrepresentation of hospital samples and lack of data on South Asian populations who exhibit a distinct central adiposity phenotype that could influence CNN performance. We quantitatively synthesised diagnostic accuracy metrics for eligible studies using random-effects meta-analyses.

**Findings:**

Our search returned 289 studies, of which 17 were eligible. All but one of the 17 studies were based in hospital or clinical outpatient settings with curated cases and controls. Studies were conducted exclusively in East Asian, European, or North American populations. Studies employed varying gold standards: seven studies (41.18%) used liver biopsy, three (17.64%) used MRI proton density fat fraction, and seven (41.18%) used clinician-evaluated ultrasound-based HS grades. The APCAPS sample included 219 participants with radiologist-assigned HS grades. Across the range of study settings and populations, CNNs demonstrated good diagnostic accuracy. Meta-analysis of studies with low risk of bias reporting on five unique datasets showed a pooled area under the receiver operating characteristic curve of 0.93 (95% CI 0.73–0.98) for detecting any severity and 0.86 (95% CI 0.77–0.92) for detecting moderate-to-severe HS severity grades, respectively.

**Interpretation:**

CNNs have good diagnostic accuracy and generalisability for HS classification, suggesting potential for real-world application.

**Funding:**

10.13039/501100000265Medical Research Council, UK (MR/T038292/1, MR/V001221/1).

**Research in context**

**Evidence before this study**

Convolutional Neural Network (CNN) algorithms have become the standard for computer vision tasks such as image classification. Recently, there has been considerable interest in applying CNNs to medical image evaluation tasks, including the ultrasonographic evaluation of hepatic steatosis (HS). An initial scoping search (in November 2023) revealed six published systematic reviews in the broader field of machine learning (ML) in hepatology, which included a variety of tasks (diagnostic, therapeutic, and prognostic modelling of HS and other liver diseases), model inputs (various imaging modalities such as CT, MRI, raw radiofrequency or quantitative or B-mode ultrasounds, and textual or structured electronic health record data), and model types (CNNs and simpler traditional approaches to image-feature extraction). These reviews demonstrated that ML-assisted systems have significant potential for application in chronic liver diseases but did not specifically try to synthesise evidence on the diagnostic performance of CNNs for classifying HS from B-mode ultrasound images. To understand the generalisability of CNNs for this specific task it is necessary to study model performances across multiple diverse datasets. Therefore, we conducted a systematic search of Ovid-MEDLINE and Embase databases up to Dec 12, 2023, for primary studies which developed or validated CNN-based algorithms for classifying HS from B-mode ultrasound images.

**Added value of this study**

As far as we know, this review is the first to systematically study the diagnostic capabilities of CNNs for the classification of HS using B-mode ultrasound imaging. We chose B-mode ultrasound imaging because it is safe, relatively inexpensive, and widely accessible. When reviewed by medical experts, this modality offers comparable accuracy to that of CT or MRI in detecting moderate-to-severe histological-grade HS. We excluded studies using ultrasound data types like raw radiofrequency or quantitative ultrasound, as these are unavailable in routinely used clinical scanners and require advanced technical expertise. This ensures our review is directly relevant to ML applications in low-resource clinical settings. Our review revealed a significant underrepresentation of community-based studies and a lack of data on south Asian populations. Hospital-recruited individuals often differ in disease characteristics from those in community settings and may not appropriately represent the full spectrum of disease presentation. This spectrum bias could potentially overestimate the performance of medical imaging prediction algorithms in certain populations. Furthermore, south Asians, who constitute a substantial global population, are shown to have a distinct phenotype characterised by increased visceral adiposity, including HS. It is currently not known whether CNN models have comparable diagnostic performance in these populations. We addressed these research gaps by supplementing our review with validation of a popular pre-trained CNN algorithm using data from the Andhra Pradesh Children and Parents’ Study (APCAPS), a large community-based cohort from South India. Across datasets with varying ethnicities and both hospital and community-based settings studied, CNNs demonstrated good diagnostic performance compared to clinical gold standards. The overlapping confidence intervals of the reported performance evaluation metrics between different populations and across CNN model architectures lend credibility to their generalisability and potential for real-world application. We then performed a meta-analysis only including studies with a low risk of bias or applicability concerns as per the Quality Assessment of Diagnostic Accuracy Research—2 tool. The strong pooled diagnostic performance metrics, with an area under the receiver operator curve of 0.93 (0.73, 0.98) for detecting any severity and 0.86 (0.77, 0.92) in detecting moderate-to-severe HS severity grades, provide a benchmark for how well these models could be expected to perform in real-world applications.

**Implications of all the available evidence**

We report favourable diagnostic performances of CNNs across diverse datasets differing in study populations (age, sex, ethnicities, and disease severities) and study settings (hospital and community-based across several countries utilising different reference standards). This lends credibility to the generalisability of this class of algorithms for the HS classification task and underscores their potential for real-world clinical application. However, we also highlight existing constraints in methodological approaches and study reporting quality. Future research should focus on the practical implementation challenges of integrating CNNs into clinical workflows and should perform recurrent local validations of their diagnostic accuracy and reliability over time in real-world settings.

## Introduction

Hepatic steatosis (HS), also referred to as fatty liver, is characterised by excessive intracellular fat accumulation in the liver.^1^^,^^2^ Prolonged HS increases the risk of liver cell injury, leading to inflammation, fibrosis, cirrhosis and its sequelae, including hepatic cancers.^1^^,^^2^ HS is the hallmark pathological feature of non-alcoholic fatty liver disease (NAFLD),^1^ which is the most prevalent cause of chronic liver disease estimated to affect nearly 1 out of every 3 adults globally.^3^ In addition to clinical corroboration, NAFLD diagnosis requires the histopathological or radiological evidence of HS.^1^^,^^2^^,^^4^

The traditional liver biopsy with histopathological assessment is regarded as the reference standard for demonstrating HS.^1^^,^^2^ However, given its invasiveness, non-invasive medical imaging techniques are becoming popular.^1^^,^^2^^,^^5^ Radiation exposure is a significant downside of computed tomography (CT).^1^ High cost restricts the utility of magnetic resonance imaging (MRI).^2^^,^^5^ Expert-reviewed ultrasound has accuracies comparable to CT or MRI for detecting moderate-to-severe histological-grade HS.^5^, ^6^, ^7^, ^8^ Additionally, ultrasounds are safe, relatively inexpensive, and broadly accessible.^1^^,^^2^^,^^5^, ^6^, ^7^ However, the need for skilled radiologists to interpret ultrasound scans can impede medical imaging service delivery.^9^ Automated medical image evaluation with machine learning (ML) tools has the potential to mitigate this issue and improve healthcare access.^9^^,^^10^ ML tools like Convolutional Neural Networks (CNNs) have demonstrated diagnostic performance equivalent to healthcare professionals across medical imaging tasks.^11^ In their ability to learn inherent spatial patterns within data, these algorithms are uniquely suited for computer vision tasks such as extracting (medical) image features for downstream analyses.^12^^,^^13^ Several studies have demonstrated favourable performances of CNNs in classifying HS from B-mode ultrasound images.^14^, ^15^, ^16^, ^17^ However, to understand their generalisability for this task, it is necessary to study their performance systematically across several diverse datasets.^18^ Previous research has largely focussed on hospital-based populations,^14^, ^15^, ^16^, ^17^ who may not capture the full spectrum of disease presentations seen in community settings, potentially inflating model performance.^19^ Conspicuously, South Asians, a substantial global population with a distinct phenotype marked by increased visceral adiposity, including HS,^20^^,^^21^ remain underrepresented, raising ethical concerns around potential racial or ethnic bias in ML algorithms.^22^ Previous systematic reviews we identified were in the broader field of ML in hepatology,^23^, ^24^, ^25^, ^26^, ^27^, ^28^, ^29^ which highlighted the significant potential of ML-assisted systems in chronic liver diseases (including HS) across diagnostic, therapeutic, and prognostic tasks. However, none specifically examined the diagnostic performances or generalisability of CNNs in classifying HS from B-mode ultrasound images.

We aimed to provide a comprehensive synthesis of the diagnostic performance and generalisability of the CNN class of algorithms for HS classification tasks using B-mode ultrasound imaging. Therefore, we conducted a systematic review and meta-analysis of the diagnostic performances of CNNs for classifying HS through B-mode ultrasound imaging, supplemented with a novel validation analysis from an underrepresented population. We fine-tuned a popular pre-trained CNN algorithm,^30^^,^^31^ using data from the Andhra Pradesh Children and Parents' Study (APCAPS), a large community-based cohort from South India.^32^ To explore model transportability between settings, we also report this APCAPS-trained model's diagnostic performance on a demographically distinct dataset by Byra and colleagues.^17^

## Methods

### Literature search and data extraction

We included studies that used a CNN to classify HS grades using conventional B-mode ultrasound images as input in human participants of any age. We excluded studies using other ultrasound data like raw radiofrequency or quantitative ultrasound (qUS) modalities, because these require dedicated hardware or software, are often proprietary, and demand advanced technical expertise for their determination.^6^^,^^33^ Consequently, they are unavailable in routinely used clinical scanners^6^^,^^33^ and are less relevant to low-resource settings. We only included studies that used liver biopsy, MRI proton density fat fraction (PDFF), or clinician-evaluated liver ultrasounds as the gold standard, as these are widely recognised diagnostic modalities for HS.^1^^,^^2^^,^^5^ Original research articles (with full-text available in English) published in peer-reviewed journals were included. Full inclusion and exclusion criteria using the population, intervention, comparison, and outcome approach are provided in Table 1.

**Table 1:** Systematic review selection criteria using the population, intervention, comparator, and outcome approach.

Population	• Humans, general population without clinically diagnosed fatty liver disease (FLD) • Humans with non-alcoholic FLD or alcohol associated FLD or those clinically deemed to be of high risk of either of them. • Participants of both sexes (male and female) across all age groups
Intervention	• Conventional B-mode ultrasound imaging of the liver—all radiological views and scanning planes • Any of the following gold standards for the diagnosis of fatty liver disease status in participants: (a) Grading of the liver ultrasounds by qualified clinicians or radiologists (b) MRI calculated proton density fat fraction (PDFF) values for quantification of hepatic steatosis (c) Liver biopsy for quantifying the % of hepatocytes showing steatosis • Convolutional neural networks for B-mode ultrasound image feature extraction and classification
Comparator	• Compare evaluation metrics (described under outcomes) between different convolutional neural network architectures
Outcome	• Evaluation metrics calculated via k-fold cross-validation or hold-out (validation or test) sets: (a) Area under the receiver operating characteristic curve, or (b) (sensitivity and specificity) for convolutional neural network-based image classification for either (a) multi-class target (Normal, Grade 1, Grade 2, or Grade 3 fatty liver disease) or (b) binary target (any binarisation of the multi-class categories)

No restrictions on study designs were applied.

We searched Ovid-MEDLINE All and Embase for studies published in English up to December 12, 2023, using search terms based on three concepts: deep learning, HS, and ultrasound imaging (full search strategy in Supplementary Information p2). The reference lists of included studies and relevant review articles were manually searched. Reviewers AJ and CA independently screened titles and abstracts of all studies and reviewed the full text when inclusion was doubtful. Conflicts were resolved through consensus reached via discussion or referral to reviewers PM and SK (Supplementary Information p3). Study data including pre-specified primary outcome measures, area under the receiver operating characteristic curve (AUC), sensitivity, and specificity values for two binary classification tasks, either any severity HS (S1, S2, or S3 grades) vs absence (steatosis grade 0, S0), or moderate-to-severe HS (S2/S3) vs normal-to-mild HS (S0/S1) were extracted using a predefined data collection form. AJ and CA independently assessed the risk of bias and applicability concerns for included studies using the Quality Assessment of Diagnostic Accuracy Research (QUADAS-2) tool adapted to evaluate studies on artificial intelligence.^23^^,^^24^^,^^34^ Our systematic review and meta-analysis protocol was registered on PROSPERO (CRD42024501483) and reported according to PRISMA guidelines.^35^

### APCAPS liver ultrasound data and processing

The details of the APCAPS population have been described elsewhere.^32^ Briefly, it is a prospective, inter-generational cohort based in the 29 villages of Ranga-Reddy district in the South Indian state of Telangana. For this analysis, we used a subset of participants aged ≥45 years (N = 2057) at the time of the last follow-up during 2022–2023.^36^

**Fig. 1:**
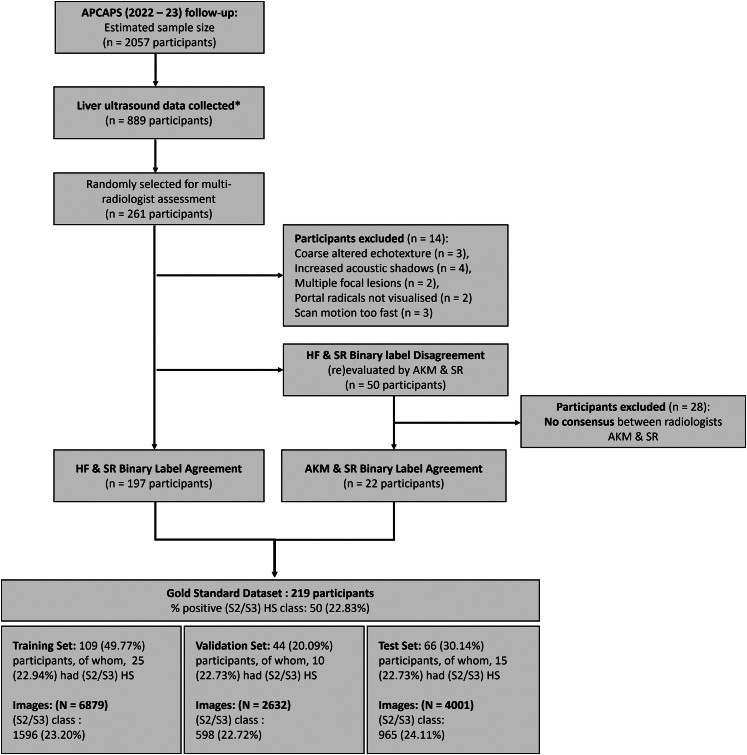
Andhra Pradesh Children and Parents' Study (APCAPS) liver ultrasound gold standard dataset generation. The binary label refers to (S2/S3) vs (S0/S1). Reported N for images refers to numbers before data augmentation. ∗As of August 2022.

The APCAPS liver ultrasound scanning protocol was designed considering the constraints around practical data acquisition in real-world settings. Mainly, we focused on settings where an automated radiological diagnosis could be beneficial. Recognising that such settings often lack skilled radiologists, we developed a straightforward, single-view (intercostal) scanning procedure that non-specialist operators, including community health workers or technicians, could readily learn (Supplementary Information p4). Using this protocol, each participant contributed a single 3–5 s video clip, yielding an average of 61 ± 17 images. Among participants with available data as of August 2022 (n = 889/2057), a random subsample of 261 was selected for expert review (Fig. 1). This sample size was determined based on resource constraints and was deemed sufficient as it exceeded that of most other studies in the field, which typically included at most 240 participants.^14^^,^^17^^,^^37^, ^38^, ^39^, ^40^, ^41^, ^42^, ^43^, ^44^, ^45^, ^46^ A gold standard binary label, either normal-to-mild HS (S0/S1; with S0 indicating no HS) or moderate-to-severe HS (S2/S3) was only assigned when there was independent agreement between two blinded radiologist evaluators (Supplementary Information p4). Expert-reviewed ultrasound and other modalities may distinguish HS into finer four-category severity grades. However, this level of granularity offers limited value outside of epidemiological research, particularly for patient management or prognosis.^47^^,^^48^ Ultimately, 219 participants were included in our gold standard dataset. The initial inter-reader agreement calculated among the 247 participants with adequate-quality scans between independent radiologist graders (HF and SR) for the binary labelling of (S2/S3) vs (S0/S1) HS, was moderate, *k* = 0.44 (Supplementary Information p4). This is consistent with estimates from routine clinical care ultrasound assessments^16^ and previous CNN validation studies.^41^ These agreement levels likely reflect the variability in scanner settings and the inherent subjectivity of visual grading.^16^ We then partitioned the dataset into three subsets (training, validation for hyperparameter optimisation, and test for evaluation metric calculation), ensuring similar proportions of classes in each subset. We performed this partition at the participant level to prevent data leakage.^49^

During training and validation, each image was treated as independent, with the participant DICOM label applied to each constituent image. Similar to previous studies,^14^, ^15^, ^16^, ^17^^,^^37^^,^^38^^,^^40^^,^^41^^,^^43^^,^^45^^,^^46^^,^^50^ this approach served as a form of data augmentation thought to help improve model generalisability. During testing, we calculated the classification probability for each image separately and derived the ensembled probability at the participant level by averaging. Evaluation metrics were calculated using the predicted probability threshold corresponding to the highest Youden index on the receiver operating characteristic (ROC) curve. For details on the image processing and fine-tuning of the pre-trained InceptionResNetV2 model for the APCAPS liver ultrasound dataset refer Supplementary Information p5-6. The protocol and tools for the APCAPS 2022-23 follow-up were approved by the ethics committees of ICMR-NIN (CR/2/II/2024) and Indian Institute of Public Health Hyderabad (IIPHH/TRCIEC/189/2018), India, and the London School of Hygiene and Tropical Medicine (21771/RR/19113), UK. All participants provided written informed consent (or thumbprint for people who did not have a high school diploma or equivalent [possibly due to opportunity gap]) to participate and for their data to be used for research purposes. Data used for analysis were fully anonymised.

### Data synthesis and statistical analysis

For each eligible study, pre-specified data, including participant demographics (sample size and age), study setting or country, ultrasound-acquisition details, model-development and validation procedures, and reported performance metrics (AUC, sensitivity, and specificity) with their 95% CIs, were extracted and collated in tables. For each binary classification task (any severity HS vs absence, and moderate-to-severe HS vs normal-to-mild HS) we performed a quantitative synthesis after excluding studies with a high or uncertain risk of bias or applicability concerns as per the QUADAS-2 tool.^34^ For outcomes that met the pre-defined criterion of being reported in at least five studies, we fitted random effects meta-analysis models to pool logit-transformed evaluation metrics and present the results in forest plots (Supplementary Information p7). We also provide funnel plots for visualisation of publication bias and small-study effects for all outcomes.

We report our APCAPS-trained model's evaluation metrics on the APCAPS test set (internal-hold-out validation) as well as on the open-source dataset by Byra and colleagues,^17^ both with additional fine-tuning (internal-hold-out validation), and without (external-hold-out validation). Confidence Intervals (CI) around AUC metrics were determined by the non-parametric DeLong's method,^41^ and those around sensitivity and specificity were obtained from true positive, true negative, false positive, and false negative, algebraically.^51^ We quantified model calibration by reporting Brier scores. For model explainability, we provide class activation map (CAM) plots.^52^

### Role of the funding source

The funders played no role in the study design, data collection, analysis, interpretation, or report writing.

## Results

**Fig. 2:**
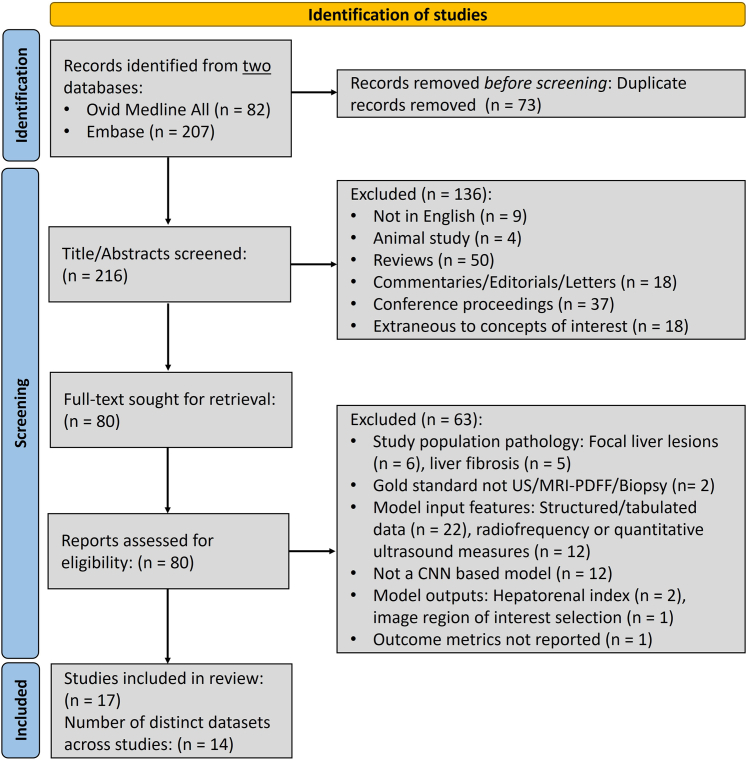
Study selection. Numbers are accurate as of Dec 12, 2023. US: Ultrasound, MRI-PDFF: Magnetic Resonance Imaging Proton Density Fat Fraction.

A total of 289 studies were identified in the initial search. Following the exclusion of duplicates, title and abstract screening, and full-text eligibility assessment, 17 studies (Supplementary Information p8-14), conducted on 14 distinct datasets, met the selection criteria (Fig. 2). Key summary statistics for the studies included in the review are provided in Table 2. Seven studies used liver biopsy as the gold standard,^16^^,^^17^^,^^37^, ^38^, ^39^, ^40^, ^41^ three used MRI-PDFF,^14^^,^^42^^,^^43^ and seven used clinician-evaluated ultrasound grades.^15^^,^^44^, ^45^, ^46^^,^^50^^,^^53^^,^^54^ All but one study^54^ were performed in a hospital or outpatient clinical setting, with specific recruitment of cases (patients with NAFLD or alcohol-associated fatty liver disease) and controls (participants without these conditions). Geographically, studies were exclusively conducted in East Asian,^15^^,^^16^^,^^40^^,^^42^^,^^44^^,^^46^^,^^50^^,^^53^^,^^54^ European,^17^^,^^37^, ^38^, ^39^^,^^45^ or North American populations.^14^^,^^41^^,^^43^ Studies included total populations ranging from 16 to 3158, with most, 12 (70.59%), including <250 participants.^14^^,^^17^^,^^37^, ^38^, ^39^, ^40^, ^41^, ^42^, ^43^, ^44^, ^45^, ^46^ Three studies trained their models on relatively large numbers of participants, ranging from 742 to 2899, while reporting evaluation metrics on hold-out sets that included 112–418 participants.^15^^,^^16^^,^^54^ Two studies failed to report complete details regarding participant numbers.^50^^,^^53^ The open-source dataset by Byra and colleagues^17^ with 55 participants was utilised in five studies, either alone^17^^,^^37^^,^^38^ or in conjunction with private datasets.^50^^,^^53^

**Table 2:** Summary statistics of key information for studies included in the systematic review.

Study characteristics	Summary statistics
Total number of studies included in the review^14^, ^15^, ^16^, ^17^^,^^37^, ^38^, ^39^, ^40^, ^41^, ^42^, ^43^, ^44^, ^45^, ^46^^,^^50^^,^^53^^,^^54^	*N* = 17
Number of distinct datasets used for analysis	n = 14
**Study sites—geographical regions**	
East Asia^15^^,^^16^^,^^40^^,^^42^^,^^44^^,^^46^^,^^50^^,^^53^^,^^54^	9 (52.94)
Europe^17^^,^^37^, ^38^, ^39^^,^^45^	5 (29.41)
North America^14^^,^^41^^,^^43^	3 (17.64)
**Study setting**	
Hospital or outpatient clinic^14^, ^15^, ^16^, ^17^^,^^37^, ^38^, ^39^, ^40^, ^41^, ^42^, ^43^, ^44^, ^45^, ^46^^,^^50^^,^^53^	16 (94.12)
Community^54^	1 (5.88)
**Gold standard methods**	
Liver histopathology^16^^,^^17^^,^^37^, ^38^, ^39^, ^40^, ^41^	7 (41.18)
MRI-PDFF^14^^,^^42^^,^^43^	3 (17.65)
Radiologist assigned US HS grades^15^^,^^44^, ^45^, ^46^^,^^50^^,^^53^^,^^54^	7 (41.18)
Total study population	90 (55–205)
Number of participants on whom evaluation metrics are reported^a^	55 (24–135)
**Ultrasound—data acquisition reporting**	
Sufficient description including scanning planes or views, and ROIs^14^^,^^16^^,^^42^^,^^43^^,^^54^	5 (29.41)
Some or no description but insufficient to facilitate replication^15^^,^^17^^,^^37^, ^38^, ^39^, ^40^, ^41^^,^^44^, ^45^, ^46^^,^^50^^,^^53^	12 (70.59)
**Number of ultrasound images per participant**	
Multiple^14^, ^15^, ^16^, ^17^^,^^37^^,^^38^^,^^40^, ^41^, ^42^, ^43^^,^^45^^,^^46^^,^^50^^,^^53^^,^^54^	15 (88.24)
Single^39^^,^^44^	2 (11.76)
**Derivation of multiple images per participant**	
Several distinct ultrasound views were acquired	
A single image per view was used^14^^,^^42^^,^^54^	3 (17.65)
Several images per view was used^16^^,^^43^	2 (11.76)
A single ultrasound view was acquired	
A set of 10 consecutive images was chosen from all acquired images^17^^,^^37^^,^^38^^,^^50^^,^^53^	5 (29.41)
A single ROI (or image patch) was manually chosen from each of the 5 acquired images^40^	1 (5.88)
Several non-overlapping ROIs (or image patches) were generated from acquired image/s^45^^,^^46^^,^^50^	3 (17.65)
Unclear^15^^,^^41^^,^^53^	3 (17.65)
**Processing of multiple image (or image patch) inputs per participant**	
Each image (or image patch) was treated as independent: during training the participant label was applied to each constituent image (or patch), and during validation/testing, the classification probability for each image (or patch) was calculated separately^14^, ^15^, ^16^, ^17^^,^^37^^,^^38^^,^^40^^,^^41^^,^^43^^,^^45^^,^^46^^,^^50^	12 (70.59)
The two images were concatenated into a single input for the CNN model^54^	1 (5.88)
Features from two images were independently extracted using separate CNN models, concatenated, and then input into a classification model^42^	1 (5.88)
**Image pre-processing**	
Cropping out machine annotations^14^^,^^16^^,^^17^^,^^37^, ^38^, ^39^^,^^41^, ^42^, ^43^	9 (52.94)
Semantic segmentation of ROIs^53^	1 (5.88)
Histogram equalisation techniques^15^^,^^53^	2 (11.76)
Image denoising (Gaussian filters)^15^	1 (5.88)
Image enhancement methods involving local phase and radial symmetry image feature extractions^38^	1 (5.88)
**Data augmentation**^b^	
Yes—Offline methods^37^^,^^38^^,^^40^^,^^42^^,^^45^^,^^46^	6 (35.29)
Yes—On-the-fly methods^14^^,^^15^^,^^53^	3 (17.65)
Did not perform data augmentation^16^^,^^17^^,^^39^^,^^41^^,^^43^^,^^44^^,^^50^^,^^54^	8 (47.05)
**CNN model architectures**^c^	
Deep Networks	
ResNet-module inspired off-the-shelf or custom deep networks^14^, ^15^, ^16^^,^^37^^,^^38^^,^^54^	6 (35.29)
Inception^37^^,^^39^^,^^45^^,^^53^	4 (23.53)
Inception-ResNet^17^^,^^37^	2 (11.76)
VGG networks^40^, ^41^, ^42^	3 (17.65)
EfficientNet^50^	1 (5.88)
Shallow (3–10) layer networks^44^^,^^46^	2 (11.76)
Unclear^43^	1 (5.88)
CNN model training leveraged transfer learning strategies^d^ (Yes)^14^^,^^15^^,^^17^^,^^37^^,^^38^^,^^40^, ^41^, ^42^, ^43^^,^^45^^,^^50^^,^^53^	12 (70.59)
**Classification models**	
Fully connected neural network^15^^,^^38^, ^39^, ^40^, ^41^, ^42^^,^^44^, ^45^, ^46^^,^^50^^,^^53^^,^^54^	12 (70.59)
Support Vector Machine^17^^,^^37^	2 (11.76)
Logistic Regression^14^	1 (5.88)
Unclear^16^^,^^43^	2 (11.76)
**Validation—methods**^e^	
Internal-hold-out^15^^,^^37^^,^^38^^,^^40^^,^^41^^,^^43^, ^44^, ^45^, ^46^^,^^53^^,^^54^	11 (64.71)
Internal-cross-validation	
5-fold^42^	1 (5.88)
10-fold^39^^,^^50^	2 (11.76)
LOOCV^14^^,^^17^^,^^38^	3 (17.65)
External-hold-out^16^	1 (5.88)
**Validation—data leakage**	
Unable to exclude leakage between train and test (hold out or CV folds)^37^^,^^44^, ^45^, ^46^^,^^50^^,^^53^	6 (35.29)
**Evaluation metrics**	
Reported at^f^	
Participant-level^14^^,^^16^^,^^17^^,^^38^, ^39^, ^40^^,^^42^^,^^44^^,^^54^	9 (52.94)
Constituent image (or image patch) level^15^^,^^37^^,^^38^^,^^41^^,^^43^^,^^45^^,^^46^^,^^50^^,^^53^	9 (52.94)
S0 vs (S1 or higher) classification^g^	
Studies reporting AUCs^14^, ^15^, ^16^, ^17^^,^^37^, ^38^, ^39^, ^40^, ^41^, ^42^^,^^44^^,^^45^^,^^54^	13 (76.47)
Studies reporting AUC with CIs^16^^,^^38^^,^^40^^,^^41^	4 (23.53)
Studies reporting sensitivity and specificity^14^^,^^15^^,^^17^^,^^37^, ^38^, ^39^, ^40^, ^41^, ^42^, ^43^^,^^45^^,^^54^	12 (70.59)
Studies reporting sensitivity and specificity with CIs^43^	1 (5.88)
(S0/S1) vs (S2/S3) classification^g^	
Studies reporting AUCs^15^^,^^16^^,^^40^^,^^41^^,^^44^^,^^54^	6 (35.29)
Studies reporting AUC with CIs^16^^,^^40^^,^^41^	3 (17.65)
Studies reporting sensitivity and specificity^15^^,^^40^^,^^41^^,^^54^	4 (23.53)
Studies reporting sensitivity and specificity with CIs	0 (0.00)
**Model explainability or interpretability**	
Class Activation Mapping Plots^14^^,^^41^^,^^42^^,^^50^	4 (23.53)
SHAP values across input image pixels^42^	1 (5.88)
Auxiliary neural network that mapped the ultrasound image to multi-class diagnostic features^54^^,^^h^	1 (5.88)

AUC: Area under the receiver operating characteristic curve, CI: Confidence Interval, CNN: Convolutional Neural Network, CV: Cross Validation, LOOCV: Leave One Out Cross Validation, MRI-PDFF: Magnetic Resonance Imaging Proton Density Fat Fraction, ROI: Region of Interest, US HS: Ultrasound Hepatic Steatosis, S0–S3: Hepatic steatosis severity grades 0–3, SHAP: Shapley additive explanations, VGG: Visual Geometry Group, vs: versus.

a Where applicable, these metrics correspond to the number of participants in the hold-out test set, or total number of participants in the dataset (for those reporting using k-fold cross-validation metrics); for studies that exclusively reported only the proportion of all images used as hold-out set, we use this proportion to calculate number of participants in the hold-out.

b When employed data augmentation techniques commonly involved flips, rotations, translations, zooms, crops, the addition of noise, or the generation of multiple non-overlapping image patches; on-the-fly refers to real-time processing where augmented images are generated during the training process whereas offline refers to pre-processing, where augmented images are generated and saved on disk before the training process.

c In one study^37^ multiple deep CNN networks were used for feature extraction, and the features were concatenated before being input into a classification model.

d By utilising a pre-trained CNN (base model) that achieved state-of-the-art multi-class classification performance (>90% top-1 accuracy) in the 1000-class image classification global ImageNet competition.

e In one study^38^ both internal-hold-out test and internal-cross-validation was reported.

f In one study^38^ metrics were reported both at the participant and image-level, however, they were reported on different partitions of the data.

g Studies reporting CIs around sensitivities and specificities did not report how they were calculated, while two^16^^,^^41^ of the four studies reporting CIs around AUCs calculated them using the DeLong's method.

h Like increased liver echogenicity, intrahepatic duct blurring, and impaired visualisation of the diaphragm.

A range of ultrasound scanners was used across studies (Supplementary Information p15) though studies infrequently reported their ultrasound scanning acquisition protocols in sufficient detail to allow for replication. Studies employed a variety of standard ultrasound imaging views, and three included within-study comparisons of CNN diagnostic performances across views.^14^^,^^16^^,^^42^ Two reported some evidence suggesting the superiority of specific views,^14^^,^^42^ while the largest of the three found statistically similar diagnostic performance and reliabilities across multiple imaging views.^16^ Notably, in 12 studies (70.59%), we observed that radiology personnel would be required to run predictions using an already trained CNN model. This radiological expertise would be needed to acquire participant images from multiple distinct scanning views,^14^^,^^16^^,^^42^^,^^43^^,^^54^ to select a sequences of images from the set of all acquired images,^17^^,^^37^^,^^38^ or for the manual delineation of region of interests (ROIs).^39^^,^^40^^,^^44^^,^^50^ We couldn't comment on the remaining five (29.41%) studies due to insufficient reporting.^15^^,^^41^^,^^45^^,^^46^^,^^53^

All^14^^,^^15^^,^^17^^,^^37^, ^38^, ^39^, ^40^, ^41^, ^42^, ^43^, ^44^, ^45^, ^46^^,^^50^^,^^53^^,^^54^ but one study^16^ reported internal (in-sample, or random split-sample) validation metrics^18^^,^^19^^,^^55^ using random split(s) of the same data pool as the training dataset (Supplementary Information p16). One study^16^ conducted internal validation but exclusively reported metrics for external (or out-of-sample) validation.^18^^,^^55^ There was a noticeable variation in the reported external validation metrics between the two datasets, attributed to lower quality of the older ultrasound scanners in one dataset.^16^ Notably, the same study also compared CNN diagnostic performances across three more recent, premium ultrasound scanners and found strong agreement across scanners.^16^ We could not comment on their model's transportability^18^ without baseline internal validation metrics. In at least 6 (35.29%) studies, we could not definitively exclude data leakage between training and test (or folds among studies using cross-validation) sets.^37^^,^^44^, ^45^, ^46^^,^^50^^,^^53^ These studies usually reported inflated evaluation metrics (Supplementary Information p17). Nearly half the studies, 8 (47.06%), failed to report evaluation metrics corresponding to the participant-level binary classifications of HS.^14^^,^^16^^,^^17^^,^^39^^,^^40^^,^^42^^,^^44^^,^^54^ Most studies, 14 (82.35%), reported evaluation metrics for the S0 vs (S1 or higher) classification task,^14^, ^15^, ^16^, ^17^^,^^37^, ^38^, ^39^, ^40^, ^41^, ^42^, ^43^, ^44^, ^45^^,^^54^ while fewer, 6 (35.29%) reported on the (S0/S1) vs (S2/S3) classification task.^15^^,^^16^^,^^40^^,^^41^^,^^44^^,^^54^ While most studies focussed on binary classification tasks, only 4 (23.53%) reported four-grade multi-class outputs,^15^^,^^46^^,^^50^^,^^53^ and the largest of these achieved per-grade AUCs ranging from 0.97 to 0.98.^15^ Studies rarely provided the associated standard errors or CIs around reported evaluation metrics. No study reported calibration plots or metrics. Among those that reported sensitivity or specificity, none reported the thresholds used for classification, while few noted that the threshold was selected to maximise Youden's index.^14^^,^^17^^,^^40^ One included study^16^ compared CNNs applied to routine B-mode images with the commercially available qUS modality controlled attenuation parameter (CAP by *FibroScan*) and found the CNN performance matched or outperformed CAP for each of the HS binary classification tasks, S0 vs (S1 or higher), (S0/S1) vs (S2/S3), and (S2 or lower) vs S3.

### Diagnostic performance of CNNs for identifying any severity HS

**Fig. 3:**
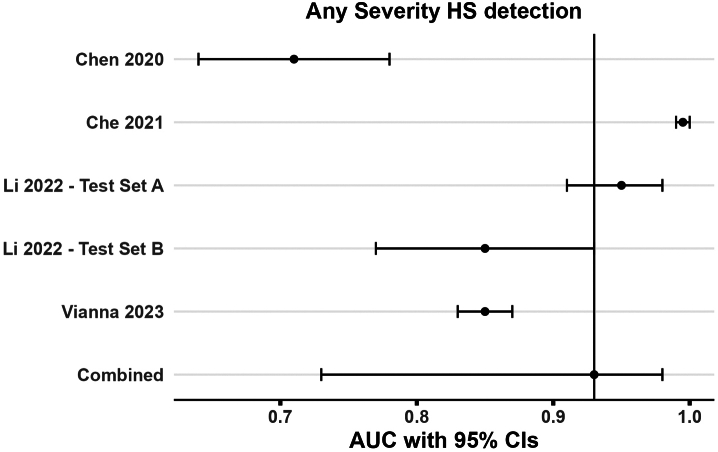
Forest plot of study datasets (n = 5) reporting AUC with 95% CIs for any severity HS detection task (S1 or higher) vs S0 HS, included in the meta-analysis. AUC: Area under the receiver operating characteristic curve, HS: Hepatic Steatosis.

Of the 17 included articles in the review, only the nine (52.94%) articles deemed to be of low risk of bias and applicability concerns (Supplementary Information p18-20) plus the novel validation analysis using APCAPS dataset were considered for quantitative synthesis (*N* = 10, Table 3). Among these ten, four studies^16^^,^^38^^,^^40^^,^^41^ reported AUC with CIs on five distinct datasets for any severity HS identification task, (S1 or higher) vs (S0), including 407 (unseen) participants. The weighted prevalence of gold standard-defined HS (S1 or higher) across studies was 72.28% (n = 294). The pooled AUC of CNN-based algorithms to detect HS, i.e., (S1 or higher) vs S0, from B-mode liver ultrasound images was 0.93 (0.73, 0.98) indicating a strong discrimination between classes (Fig. 3). We noted strong evidence for inter-study statistical heterogeneity in reported metrics, *I*^2^: 99.9%, Q test p value < 10^−3^, which corroborated the observed methodological heterogeneity. Given the small sample number of studies per stratum, we could not perform any sub-group analyses.

**Table 3:** Characteristics of low risk of bias studies included in the quantitative synthesis (n = 10 studies).

Sr	Study/year	Study population (Setting/Country, FLD/total, Steatosis grades, BMI)		Ultrasound scan protocol & ROIs	Total number of images in ground truth dataset before and after augmentation	CNN algorithm (Feature extraction + classification)^b^	Validation methods	Evaluation metrics^c^
**Ground truth: Liver Biopsy**^a^								
1.	(Byra et al., 2018)^17^	• Hospital/Poland • 38/55 • Among those with FLD: 52.63% had ≤ 35% steatosis (mild grade) • Overall Population BMI: 45.9 ± 5.6		• No specific mention of the ROIs in the liver, scanning planes or views. • 10 consecutive images from an image loop sequence from each participant were used—no mention of how this sequence was chosen from all images in the patient's DICOM. • US images included both the liver and kidney	• 550 (380 FLD, 170 normal) • Did not perform data augmentation	• Pretrained Inception-ResNet-v2 + SVM	• Participant-specific LOOCV producing training and test sets (Internal-cross-validation)	**(S1 or higher) vs (S0) HS**: AUC: 0.977 ± 0.021, Sensitivity: 100%, Specificity: 88.20%
2.	(Che et al., 2021)^38^				• 550 (380 FLD, 170) • Augmented^d^ to 2000 (1000 FLD, 1000 normal)	• Images combined with their local phase filtered image and radial symmetry transformed image formed multi-feature inputs to a pretrained multi-scale ResNet with mid-fusion of features • Softmax dense layers	Used two different paradigms: (1) Participant-specific LOOCV (Internal-cross-validation) (2) 30% of patients allocated to a hold-out test set: 10 FLD + 5 Non-FLD (internal-hold-out validation) with metrics were reported at the image-level.	**(S1 or higher) vs (S0) HS**: (1) CV: AUC: 1 (0.99–1) (2) Hold-out test set: Sensitivity: 97.2% Specificity: NR
3.	(Chen et al., 2020)^40^	• Hospital/Taiwan • 126/205 • 38.54% normal, 36.10% mild, 17.07% moderate, 8.29% severe • Overall population BMI: 25.3 ± 3.8		• 5-independent intercostal scans with manual physician delineated ROIs	• 1025 images^e^ • Data augmentation^d^ was performed on the training set by random cropping within the original ROIs for the infrequent class to overcome class imbalance (numbers NR)	• Pretrained VGG-16 with 3 fully connected layer classifier top with soft max activation.	20% of participants (n = 41) formed a hold-out test (internal-hold-out validation)	**(S1 or higher) vs (S0) HS**: AUC: 0.71 (0.64–0.78), Sensitivity: 73.18%, Specificity: 60%. **(S2/S3) vs (S1/S0) HS:** AUC: 0.75 (0.67–0.82), Sensitivity: 63.25%, Specificity: 74.82%.
4.	(Li et al., 2022)^16^	• Hospital/Taiwan • Development: 370/2899; Testing—A: 123/147; Testing—B: 68/112. • Testing—A: 24.49% mild, 23.81% moderate, 35.357% severe; Testing—B: 25.89% mild, 12.5% moderate, 22.32% severe. • Overall population BMI in Testing—A: 26.67, Testing—B: 26.33		• Images were acquired from four view groups: left liver lobe (longitudinal + transverse), right liver lobe (intercostal), liver-kidney contrast (lower right love intercostal + subcostal), and subcostal (with hepatic veins). • In the development cohort, a single patient, had multiple studies, and each study contributed multiple images for algorithm development. • In the testing cohorts, each patient had a single study, with each study having multiple images (across different view groups)	• 200654 images in the development cohort • No mention of data augmentation	• ResNet 18 (does not mention whether pretrained or not) used to predict a continuous score for each individual image, then ensembled by taking the mean of the image-wise scores within and across each view group for final classification at a given participant's study level.	• Participants from independent hold-out test sets without and with blinding (Testing A & B, respectively) of labels to deep learning development team. • This was a form of external-hold-out validation as participants in Testing A & B (ground truth: histopathology) came from a distinct setting from that of those participants in the development cohort (ground truth: radiologist assigned ultrasound HS grade) • Internal-hold-out or internal-cross-validation metrics are not reported. • CIs around AUCs were obtained using the DeLong method.	**(S1 or higher) vs (S0) HS**: Testing—A: AUC: 0.95 (0.91–0.98) Testing—B: AUC: 0.85 (0.77–0.93) **(S2/S3) vs (S1/S0) HS:** Testing—A: AUC: 0.92 (0.88–0.96) Testing—B: AUC: 0.91 (0.85–0.97)
5.	(Vianna et al., 2023)^41^	• Hospital/Canada • 142/199 • 43.7% mild, 12.6% moderate, 15.1% severe FLD • Overall population BMI: 30.5 ± 7.8		• Images were said to be acquired according to the institutional clinical US protocol (not described). • A single patient contributed multiple images	• 7529 images (966 normal, 3312 mild, 1683 moderate, and 1568 severe). • Did not perform data augmentation.	• Pretrained VGG-16 architecture + Softmax dense layer • No ensembling was not performed at the patient level, and predictions were obtained on all images in single tests.	• Metrics reported on at the image-level on 26% of participants (N = 52, with 12 S0, 17 S1, 11 S2, and 12 S3 grades) used as hold-out test set (internal-hold-out validation). • CIs around AUCs were obtained using the DeLong method.	**(S1 or higher) vs (S0) HS**: AUC: 0.85 (0.83–0.87), Sensitivity: 79%, Specificity: 78%. **(S2/S3) vs (S1/S0) HS:** AUC: 0.73 (0.71–0.75), Sensitivity: 76%, Specificity: 58%
**Ground truth: MRI-PDFF values**^f^								
6.	(Byra et al., 2021)^14^	• Hospital/USA • 118/135 • Among those with FLD, 95% had PDFF ≤30% • Overall population BMI: 31 ± 5	• Four distinct images per participant were used. • One each from the 3 views in the transverse plane: hepatic veins at the confluence with the inferior vena cava, right portal vein, and right posterior portal vein • One view in the sagittal plane: liver and kidney		• 135 images per view (118 FLD, 17 normal) x 4 views • Images were augmented^d^ (appears to be on-the-fly augmentation)	• Pretrained ResNet-50 + Logistic regression (or Lasso Linear regression) for each ultrasound view trained separately. • Followed by, an ensemble model, averaging the outputs of the individual models, was constructed.	• Participant-specific LOOCV producing training and test sets (internal-cross-validation)	**(S1 or higher) vs (S0) HS**: AUC: 0.91 ± 0.03, Sensitivity: 0.80 ± 0.05, Specificity: 0.88 ± 0.05
7.	(Kim et al., 2021)^42^	• Hospital/South Korea • 39/90 • Mean 11.82% ± 8.74%, and 11.49% ± 5.49% in groups without and with alcohol exposure • NR	• 2 images per participant were used. • Right intercostal view of the liver • Right intercostal view of the liver containing right renal cortex		• 90 images per view (39 FLD, 51 normal) x 2 views • Each original image was augmented^d^ to 39 images.	Features extracted from each of the two views, separately, using pretrained VGG-19, followed by feature concatenation + Sigmoid dense layer	• Metrics reported at the participant-level using 5-fold CV (internal-cross-validation)	**(S1 or higher) vs (S0) HS**: AUC: 0.87; Sensitivity: ∼70%; Specificity: 80.5%
8.	(Tahmasebi et al., 2023)^43^	• Outpatient centre/USA • 70/120 • Mean 16.1% ± 0.07% • BMI in FLD: 34.7 ± 7.4, non-FLD: 29.9 ± 7.8	• Ten distinct images per participant. • Two images from the sagittal-subxiphoid view, 1 from transverse-subxiphoid view, 2 from sagittal-intercostal view, 1 from sagittal-subcostal view, 4 from transverse intercostal view. Different images of the same view were taken at different levels.		• 1191 images (643 FLD + 548 Non-FLD) in the training set and 244 images in the hold-out test set. • No mention of data augmentation.	• Google's AutoML Vision^g^ • No ensembling was performed at the patient level, and predictions were obtained on all images in single tests.	• Metrics reported at the image-level on 20% of participants (12 with ≥S1 HS + 12 S0 HS) used as hold-out test set. (internal-hold-out validation)	**(S1 or higher) vs (S0) HS**: Sensitivity: 72.2% (63.1–80.1) Specificity: 94.6% (88.7–98.0)
**Ground truth: Ultrasound grading by radiologists**^h^								
9.	(Yang et al., 2023)^54^	• Community/China • 615/928 • 48% mild, 7.3% moderate, 11% severe. • Overall Population: 23.8 ± 3.2	• Two images per participant were concatenated and used—epigastric longitudinal scanning in the median sagittal plane in the subxiphoid region + right subcostal scanning along the right subcostal margin.		• 928 (two images from each participant were concatenated into one) • No mention of data augmentation	• Custom 2-section Neural Network with 3 ResNet inspired blocks to extract image features and predict ‘bright liver’, ‘intra-hepatic duct blurring’, ‘impaired diaphragm visualisation’, which were then concatenated • A fully connected layer for classification.	• A hold-out test set of 186 (20%) of participants (internal-hold-out validation)	**(S1 or higher) vs (S0) HS**: AUC: 0.90; Sensitivity: 88.6%; Specificity: 90.5%. **(S2/S3) vs (S1/S0) HS:** AUC: 0.84; Sensitivity: 76.%; Specificity: 92.8%.
10.	APCAPS (2024) (this study)	• Community/India • 50/219 had moderate-to-severe (S2/S3) HS • Overall Population: 22.61 ± 4.25 kg/m^2^	• Image frames from a 3–5 s ultrasound video of the oblique intercostal view of the right lobe of the liver with 5–6 degrees of angulation—each participant contributed multiple images (varying based on the length of the video)		• 6879 images from 109 (50%) participants in the training set • On-the-fly data augmentation^d^ was performed	• A pre-trained Inception-Resnet V2 with a custom classifier top • Predictions were obtained by calculating the classification probability for each image separately and deriving the ensembled probability at the participant level by averaging.	• Metrics reported at the participant-level on a hold-out test set of 66 (30.40%) of participants. (internal-hold-out validation)	**(S2/S3) vs (S1/S0) HS:** AUC: 0.90 (0.75, 1.00); Sensitivity: 80.00 (64.29, 100); Specificity: 98.03 (74.55, 100)

APCAPS: Andhra Pradesh Children and Parents' Study, AUC: Area under the receiver operating characteristic curve, BMI: Body Mass Index, CI: Confidence Interval, CNN: Convolutional Neural Network, CV: Cross Validation, FLD: Fatty Liver Disease, HS: Hepatic Steatosis, LOOCV: Leave One Out Cross Validation, ML: Machine Learning, MRI-PDFF: Magnetic Resonance Imaging Proton Density Fat Fraction, NR: Not Reported, ROI: Region of Interest, S0–S3: Hepatic steatosis severity grades 0–3, SVM: Support Vector Machine, US: Ultrasound, VGG: Visual Geometry Group, USA: United States of America, vs: versus.

a All studies using liver histopathology used ≥ 5% liver cell steatosis on biopsy for a diagnosis of S1 HS; Chen et al., 2020, Li et al., 2022 and Vianna et al., 2023 used cut-offs of 5–33%, 34–66%, >67% for mild (S1), moderate (S2), and severe (S3) grades of HS on biopsy.

b When multiple CNN algorithms were studied, only those with the highest AUC, or highest (sensitivity/specificity) are mentioned; algorithms when pre-trained were done so on the ImageNet database.

c Where applicable evaluation metrics are reported as metric (95% CI), or mean ± standard deviation of metric across cross-validation folds. The mean ± standard deviation for AUC reported without standard errors or confidence intervals could not be included in the quantitative pooling of AUC diagnostic performance metric of CNNs across studies.

d Data augmentation was performed using translations, rotations, translations, flipping, zoom (in and out), and scaling.

e This study collected ultrasound radiofrequency data but converted to B-mode images for input into CNN models for the results tabulated.

f >5% MRI-PDFF values indicated a diagnosis of FLD for Byra et al., 2021 and Kim et al., 2021; Tahmasebi et al., 2023 used a cut-of >6.4%.

g The specific implementations of the underlying model architecture is proprietary to Google and not disclosed; however, the documentation mentions it is based on Google's leading image recognition approaches including transfer learning and neural architecture search technologies—thus highly likely to be based on convolutional neural network architectures.

h Yang et al., 2023 graded FLD as: none steatosis (S0), mild (S1, based on bright liver), moderate (S2, based on S1 + intrahepatic duct blurring), and severe (S3, based on S2 + impaired visualisation of more than half of the diaphragm); APCAPS analysis graded FLD as follows: mild (S1, based on diffusely increased hepatic echogenicity but periportal and diaphragmatic echogenicity still appreciable), moderate (S2, based on diffusely increased hepatic echogenicity obscuring periportal echogenicity but diaphragmatic echogenicity still appreciable), severe (S3 based on diffusely increased hepatic echogenicity obscuring periportal as well as diaphragmatic echogenicity), normal (S0, no increase in hepatic echogenicity).

Additionally, among the ten studies considered for quantitative synthesis, seven studies,^14^^,^^17^^,^^40^, ^41^, ^42^, ^43^^,^^54^ each conducted on a distinct dataset, reported sensitivities and specificities for the same any severity HS identification task. Pooled sensitivities and specificities obtained from bivariate diagnostic modelling revealed strong discrimination between target classes, 79.30% (71.70–85.30) and 81.20% (71.40–88.20), respectively (Supplementary Information p21-22).

### Diagnostic performance of CNNs for identifying moderate-to-severe HS

Among the 10 studies with a low risk of bias considered for quantitative synthesis, three reported AUC (with associated CIs) for the detection of moderate-to-severe HS on four distinct datasets,^16^^,^^40^^,^^41^ with a total *N* = 5 including the APCAPS dataset.

**Fig. 4:**
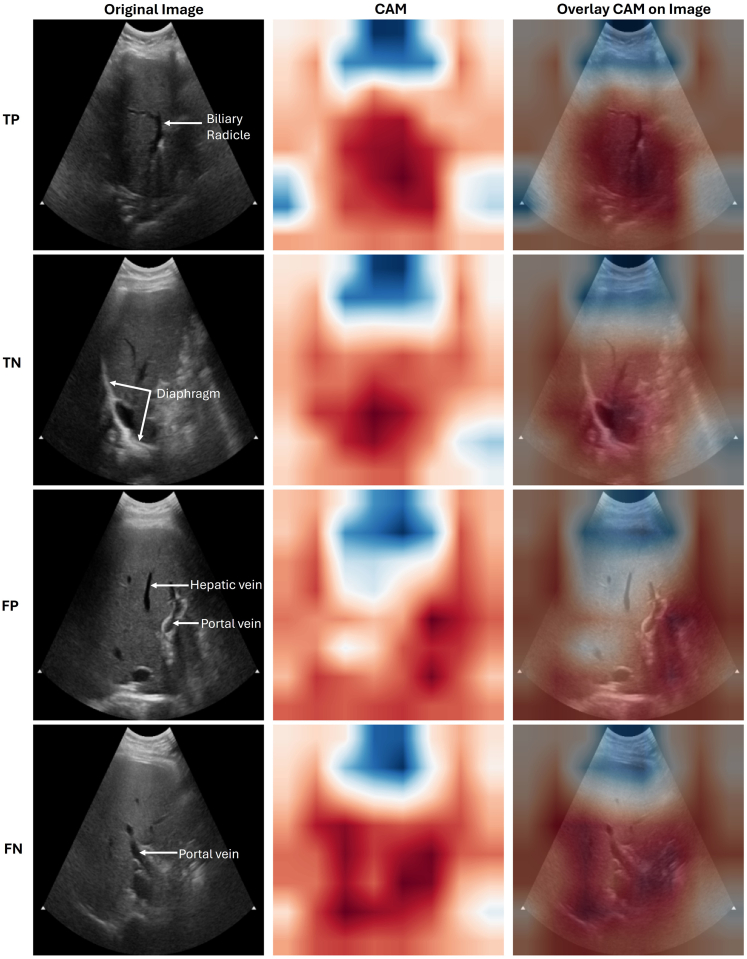
Class Activation Maps (CAM) for a randomly selected True Positive (TP), True Negative (TN), False Positive (FP), and False Negative (FN) prediction. The target class predicted is moderate-to-severe (S2/S3) HS. Red areas represent image regions that are most relevant for model prediction, with higher intensities (darker red) corresponding to higher importance. Similarly, blue areas represent regions that the model considers least relevant for prediction, with higher intensities corresponding to lower importance. Plots reveal that the model focuses on mid- and far-fields (the bottom 2/3) of the ultrasound image, primarily on the bulk of the hepatic parenchyma including the diaphragm and portal vasculature.

The APCAPS gold standard ultrasound dataset included 219 participants (aged 58.85 ± 6.69 years, 61.64% female) with a body mass index of 22.61 ± 4.25 kg/m^2^ and a prevalence of radiologist-assigned moderate-to-severe (S2/S3) HS of 22.83% (n = 50). This subsample was representative of the eligible 45+ population (N = 2057) with respect to age, sex, and body mass index distribution (Supplementary Information p23; all p > 0.05). On the APCAPs test set of 66 participants, our InceptionResNetV2 fine-tuned on the APCAPs train dataset achieved strong internal-hold-out diagnostic performance metrics in the participant-level detection of moderate-to-severe HS, i.e., (S2/S3) vs (S0/S1), AUC 0.90 (0.77, 1.00), sensitivity 80.00 (51.91, 95.67), and specificity 98.03 (89.55, 99.95) (Table 4). The model was well calibrated (Brier score: 0.10) with predicted probabilities ranging from 0.06 to 0.92. To assess the transportability of the APCAPS-trained (S2/S3) HS prediction model to a disparate clinical setting, we performed external-hold-out validation by evaluating our model's off the shelf performance in predicting (S1 or higher) HS on the demographically distinct (full) dataset by Byra and colleagues, 2018,^17^ and found a discernible drop in model performance. Calibration was markedly poorer with predicted probabilities tightly clustered between 0.14 and 0.16. However, fine-tuning the model with a 30% random subset of the Byra and colleagues, dataset,^17^ improved both its performance and calibration on the remaining 70% of the dataset. We noted improved model performance when reported at the participant level vs image level and marked variation in image level predicted probabilities for a given participant (Supplementary Information p24). CAM plots revealed that our model's predictions aligned well with the radiological criteria used for the gold standard HS assignment (Fig. 4).

**Table 4:** Participant-level evaluation performance metrics for the InceptionResNetV2 CNN model across HS classification tasks reported on the APCAPs test set (internal-hold-out validation), and the Byra and colleagues^17^ dataset both without additional fine-tuning (external-hold-out validation), and with (internal-hold-out validation).

Evaluation metrics reported on	APCAPs test set: (*N* = 66)	Full dataset by Byra et al., 2018: (*N* = 55)	A 70% random subset of Byra et al.^17^ (*N* = 39)
Validation type	Internal-Hold-Out	External-Hold-Out	Internal-Hold-Out
Base model	ImageNet pre-trained CNN		APCAPS train set pre-trained CNN
Fine tuning on	APCAPS train set		A random 30% subset of Byra et al.^17^ dataset
Prediction target class	(S2/S3) radiologist-assigned HS ultrasound grades	(S1 or above) HS by liver histopathological assessment	
Prediction threshold	0.31	0.15	0.56
Target class prevalence	22.73% (n = 15)	69.09% (n = 38)	69.23% (n = 27)
Key metrics			
AUC	0.90 (0.77, 1.00)	0.76 (0.64, 0.89)	0.84 (0.72, 0.95)
Sensitivity	80.00 (51.91, 95.67)	60.53 (44.98,76.06)	70.37 (49.82, 86.25)
Specificity	98.03 (89.55, 99.95)	88.24 (63.55, 98.54)	91.67 (61.52, 99.79)
Brier score	0.10	0.50	0.15

All metrics are reported at the participant level. AUC, Sensitivity, and Specificity are reported as point estimate followed by 95% confidence interval limits in parentheses. Brier scores range from 0 to 1, with lower values indicating better model calibration. APCAPS: Andhra Pradesh Children and Parents' Study, AUC: Area under the receiver operating characteristic curve.

**Fig. 5:**
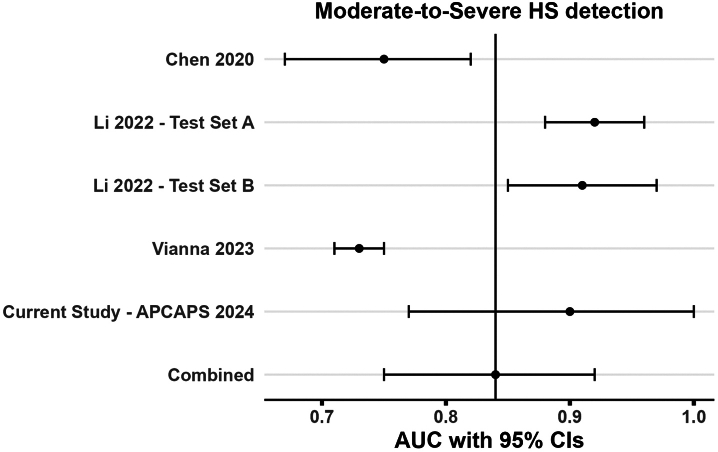
Forest plot of study datasets (n = 5) AUC with 95% CIs for moderate-to-severe HS detection task, (S2/S3) vs (S0/S1), included in the meta-analysis. AUC: Area under the receiver operating characteristic curve, CI: Confidence Interval, HS: Hepatic Steatosis.

We pooled the AUC estimates across the five datasets, including 418 (unseen) participants. The weighted prevalence of gold standard-defined moderate-to-severe HS (S2/S3) across studies was 41.72% (n = 174). The pooled AUC for CNN-based algorithms to identify moderate-to-severe HS was 0.86 (0.77, 0.92) indicating a strong discrimination between target classes (Fig. 5). We noted strong evidence for inter-study statistical heterogeneity in reported metrics, *I*^2^: 99.9%, Q test p value < 10^−3^, along with substantial methodological heterogeneity.

Funnel plots for all different examined outcomes demonstrated some asymmetry with few studies laid beyond the pseudo-95% limits (Supplementary Information p25-26).

## Discussion

We aimed to assess the diagnostic accuracy and generalisability of CNNs for classifying HS from B-mode liver ultrasounds across various settings and populations. We conducted a systematic review, supplemented with a cross-sectional analysis of the APCAPS cohort in Telangana, India. Across the range of ethnicities and hospital and community-based settings studied, CNNs demonstrated good diagnostic performance compared to currently accepted clinical gold standards. This held true despite considerable variation across studies in data acquisition methods (ultrasound scanners, scanning protocols, ROI definitions), gold standards, model architectures, and validation strategies. Our finding of consistently high point estimates and largely overlapping confidence intervals for AUCs, sensitivities, and specificities lend credibility to the generalisability of this class of algorithms for the HS evaluation task. Our meta-analysis of low-risk-of-bias studies established diagnostic performance metrics for CNNs in B-mode ultrasound-based HS classification tasks. This provides a benchmark for how well such models could be expected to perform in real-world applications.

In recent years, deep learning tools, specifically CNNs, have been shown to have diagnostic performance equivalent to that of healthcare professionals across various medical imaging classification tasks.^11^ Our meta-analysis showed CNN models for HS classification from B-mode ultrasound images have pooled AUCs ranging from 0.86 to 0.93, meeting the threshold (>0.8 at validation) commonly considered good or excellent for clinical prediction models.^56^ These figures overlap with those of widely used qUS techniques for HS classification, ultrasound-guided attenuation parameter (AUCs: 0.85–0.89) and CAP (AUCs: 0.80–0.94).^57^ However, unlike qUS, B-mode images are produced by all routinely used clinical ultrasound scanners. Thus, CNNs that analyse them offer a scalable alternative for low-resource settings. Since our search cut-off, new hospital-based^58^, ^59^, ^60^ and biobank^61^ studies, including those that adopt multi-instance learning^60^^,^^61^ (training models on all images from a participant's ultrasound examination at once, rather than treating each image as independent as done in APCAPS and most earlier studies), have reported diagnostic performances comparable to our pooled estimates. CAM plots from ours and previously published HS classification models^14^^,^^41^^,^^42^^,^^50^ suggest that CNNs focus on radiologically relevant areas of the liver, helping explain model decisions. Our findings highlight the potential for using CNN algorithms in scenarios where patient characteristics, skilled human resources, infrastructure, or costs preclude performing any of the current gold standard tests for HS classification. In high-resource settings, CNNs could be used for opportunistic screening for HS based on abdominal ultrasound scans performed for unrelated indications,^62^ or to provide real-time decision support during conventional sonography. However, despite extensive research on model development and validation, relatively few studies have examined real-world deployment of deep learning models, reflecting broader challenges.^63^ Randomised controlled trials on ML-assisted ultrasonography have shown significant time-savings and reduced sonographer cognitive overload,^64^ and improved diagnostic performance, especially when used by non-experts.^65^ Still, successful clinical translation requires addressing key issues, specifically external validation.

Consistent with previous work in deep learning for medical imaging,^11^ we found that studies rarely report externally validated metrics, raising concerns about generalisability.^18^^,^^66^ However, a singularly externally validated model may not perform consistently across varying populations, geographies, and health facilities, even for identical tasks.^66^ As with published clinical predictive models with image-^11^^,^^67^ or non-image-based^68^ inputs, we noted slightly higher internal validation diagnostic performance metrics on the APCAPS test set compared to external validation on the Byra and colleagues dataset.^17^ However, fine-tuning with just 30% of external data substantially improved performance. Whether this performance improvement stems from the merely increased training data variability or that of training data specific to the population in the evaluation set, is unanswered. This highlights a key issue in the deployment of ML models for clinical practice: models trained on one demographic (e.g., hospital-based, high-resource setting) may not transport as is to disparate settings (e.g., community-based, low-resource) due to distribution shifts in disease prevalence and presentations.^19^^,^^69^ Emerging work on federated learning offers a potential solution. By enabling model training across multiple distinct sites without centralising data, federated approaches preserve privacy while improving generalisability.^70^ Further, even within a given setting, models are prone to performance degradation over time owing to distribution drifts^66^ caused by changes in ultrasound imaging hardware/software, data acquisition protocols, or population demographics. This underscores the need for recurrent local validation to ensure reliable performance in real-world applications.^66^ Beyond good diagnostic performances, a model must also be well calibrated. That is, their output probabilities should accurately reflect the true class likelihood of the target condition in the intended clinical context.^71^

None of the studies included in our review reported calibration metrics or decision thresholds, limiting real-world clinical interpretability and implementation. During internal validation with the APCAPs test set, our model was well calibrated and produced a plausible spread of predicted probabilities. This suggests that, if deployed in this setting, the ROC-derived threshold could be meaningfully adjusted based on local prevalences, misclassification costs, or resource constraints.^72^ However, calibration was markedly poorer during external validation on the Byra et al., 2018 dataset,^17^ despite good diagnostic performance. The predicted probabilities were tightly clustered, rendering the ROC-derived threshold uninformative for clinical decision-making. Consequently, without model recalibration, any threshold adjustment to evolving clinical contexts remain limited.^72^

As far as we know, this is the first review to focus on the diagnostic utility of a specific class of ML algorithms, namely CNNs, for the granular task of evaluating HS from ultrasound scans. Including only the conventional B-mode imaging variety ensured that our review remained highly applicable to using ML tools in routine clinical settings. Studies identified predominantly involved hospital-based research conducted on White or East Asian populations, with model inference requiring the intervention of highly skilled healthcare professionals for data acquisition or subsequent ROI selection. Hospital-recruited individuals often differ significantly from community populations,^19^ potentially leading to an exaggerated dichotomy in health and disease representation. To address the limitations of the studies included in the review, our approach employed a simplified non-specialist ultrasound data acquisition protocol. Ultrasound video clips were short (up to 5 s), and neither model training nor inference required manual, domain-knowledge-driven image (or ROI) selection. We demonstrated that a pre-trained CNN, with minimal fine-tuning, can achieve satisfactory diagnostic performance in a large, community-based sample from an ethnically distinct cohort in rural South India, thus expanding the current evidence base. Moreover, we report metrics for identifying moderate-to-severe HS, a component frequently overlooked in prior studies. This is important as moderate-to-severe HS, rather than mild HS, is more strongly associated with morbidity, including the onset of diabetes resulting from impaired glucose tolerance,^73^ progression from pre-hypertension to hypertension,^74^ and adverse outcomes in coronary artery disease.^75^

The primary limitations of our study stem from the limitations of the studies included in our systematic review and meta-analysis. First, in several studies, we noted that using a trained CNN model to obtain predictions on new data would require intervention from highly skilled radiology personnel. Thus, reported performance metrics may not be representative of real-world applications since skilled personnel are often scarce in the settings where such ML models are most needed.^9^ Second, several studies lacked detailed reporting of methodology and results. Many did not specify ultrasound data acquisition details such as scanning planes, views, or specific ROIs, limiting reproducibility. While we do not recommend any particular view over another, ultrasound imaging protocols should be standardised and sufficiently detailed to enable replication and comparison. Studies that rely on expert grading for the gold standard, whether ultrasound- or histology-based, rarely report inter-reader reliability. Although low agreement in the training set labels can be mitigated by large sample sizes or noise-robust learning strategies,^76^ poor reliability in the test set labels erodes confidence in the reported evaluation metrics. Studies mostly reported model performance metrics only at the image level, which we show introduces bias. Correspondingly, the clinical decision to subject a patient to additional HS diagnostic testing is contingent on them being classed as having ultrasound features of HS and not on their constituent image's assignment. Additionally, studies rarely report standard errors (or confidence intervals) for diagnostic performance, hindering uncertainty assessment and limiting the potential for evidence synthesis across studies. Third, the substantial statistical and methodological heterogeneity, and the expected variation in the (not reported) thresholds for prediction across included studies is likely to have introduced some bias in our calculated pooled estimates. Therefore, these should not be extrapolated beyond explicitly studied contexts. Fourth, given that fewer than 10 studies were available for each outcome in our meta-analysis, it precluded statistical tests for funnel plot asymmetry for a quantitative assessment of publication or reporting biases.^77^ Qualitatively, the predominance of studies from high-income countries, selective reporting of evaluation metrics, and the paucity of studies reporting poor model performances indicate some publication bias in the current evidence base. The observed visual asymmetry in our funnel plots also supported this. Limiting the review to English-language articles may have introduced a minor additional bias, given the tendency for studies with positive results to be more frequently published in English compared to non-English languages.

Future research should design data acquisition and processing pipelines mindful of the typical resource constraints of the model's intended application settings. It should also address the practical challenges of integrating CNNs into clinical workflows and perform recurrent local validations of diagnostic accuracy and reliability over time in real-world settings. Additionally, as previously highlighted, there is a need for the standardisation of reporting in medical deep learning research.^11^

We report favourable diagnostic performances of CNNs across diverse datasets differing in study populations (age, sex, ethnicities, and disease severities) and study settings (hospital and community-based across several countries utilising different reference standards). This lends credibility to the generalisability of this class of algorithms for the HS classification task and underscores their potential for clinical application. We also highlight existing constraints in methodological approaches and study reporting quality. The current evidence justifies the need for large-scale, high-quality, longitudinal research to investigate such ML algorithms' real-world routine clinical application.

## Contributors

AJ, SK, PM conceptualised the study and prepared the systematic review protocol. SB, JL, HM, SKB curated the APCAPs data. SR, FHM, AKM annotated the ultrasound image data. AJ and CA performed the literature review. AJ conducted the formal analysis. AJ, CA, and PM have access to and have verified the data. AL performed the statistical review. SK and PM supervised the study. AJ wrote the first draft of the paper. All authors edited the subsequent drafts and approved the final version of the paper for submission.

## Data sharing statement

The APCAPS study data are available upon request to the APCAPS research co-ordinator (apcaps.crf@gmail.com), subject to approval by the APCAPS Executive Committee. The python code to obtain the APCAPS trained CNN model and run predictions on new data is available on GitHub (https://github.com/akshay-tj/fatty-liver-ultrasound-CNN.git).

## Declaration of interests

AJ and PM received research funding (salary support) from Medical Research Council, UK. We declare no other competing interests.
