# Supplementary material for: Diagnostic accuracy of convolutional neural networks in classifying hepatic steatosis from B-mode ultrasound images: a systematic review with meta-analysis and novel validation in a community setting in Telangana, India
Source: Lancet Reg Health Southeast Asia. 2025 Jul 31;40:100644. doi: 10.1016/j.lansea.2025.100644 (PMC12337209; doi:10.1016/j.lansea.2025.100644)
Supplement: Supplementary Information [file mmc1.docx]

**Supplementary Information**

**Diagnostic accuracy of convolutional neural networks in classifying hepatic steatosis from B-mode ultrasound images: a systematic review with meta-analysis and novel validation in a community setting in Telangana, India**

*Akshay Jagadeesh^a^*, Chanchanok Aramrat^a^, Santosh Rai^b^, Fathima Hana Maqsood^c^, Adarsh Kibballi Madhukeshwar^d^, Santhi Bhogadi^e^, Judith Lieber^a^, Hemant Mahajan^e^, Santosh Kumar Banjara^e^, Alexandra Lewin^f^, Sanjay Kinra^a^, Poppy Mallinson^a^*

^a^Department of Non-communicable Disease Epidemiology, Faculty of Epidemiology and Population Health, London School of Hygiene & Tropical Medicine, London, WC1E 7HT, UK
^b^Department of Radiology, Kasturba Medical College Mangalore, Manipal Academy of Higher Education, Karnataka, 576 104, India
^c^NMC Specialty Hospital, Al Ain, P.O. Box: 84142, Abu Dhabi, United Arab Emirates
^d^Yenepoya Medical College, Yenepoya (Deemed to be University), Mangaluru, Karnataka, 575 018, India
^e^Indian Council of Medical Research—National Institute of Nutrition, Hyderabad, 500007, Telangana, India
^f^Department of Medical Statistics, Faculty of Epidemiology and Population Health, London School of Hygiene & Tropical Medicine, London, WC1E 7HT, UK

* Corresponding author

Department of Non-communicable Disease, Epidemiology, Faculty of Epidemiology and Population Health, London School of Hygiene & Tropical Medicine, London, WC1E 7HT, UK.

*E-mail address:* [Akshay.Jagadeesh@lshtm.ac.uk](mailto:Akshay.Jagadeesh@lshtm.ac.uk) (A. Jagadeesh)

**Table 1: Glossary of frequently used abbreviations in the main text:**

| **Abbreviation** | **Full form** |
| --- | --- |
| APCAPS | Andhra Pradesh Children and Parents Study |
| AUC | Area Under the Receiver Operating Characteristic Curve |
| CAMs | Class Activation Maps |
| CAP | Controlled Attenuation Parameter |
| CI | Confidence Interval |
| CNNs | Convolutional Neural Networks |
| CT | Computed Tomography |
| HS | Hepatic Steatosis |
| ML | Machine Learning |
| MRI | Magnetic Resonance Imaging |
| NAFLD | Non-Alcoholic Fatty Liver Disease |
| PDFF | Proton Density Fat Fraction |
| qUS | Quantitative Ultrasound |
| QUADAS | Quality Assessment of Diagnostic Accuracy Studies |
| ROI | Region of Interest |
| ROC | Receiver Operating Characteristic |

**Systematic Review: Search Strategy**

**Table 2:** Keyword search terms for OVID MEDLINE ALL and EMBASE databases

| **Concept 1** | **Concept 2** | **Concept 3** |
| --- | --- | --- |
| artificial intelligence OR machine learning OR deep learning OR artificial neural network* OR deep neural network* OR computer vision OR gradient descent OR gradient based learning OR gradient method* OR convolutional neural network* OR CNN OR CNNs OR ConvNet* OR AlexNet OR inception module OR inception-v3 OR inception-v4 OR inception-res* OR GoogLeNet OR ResNet OR  EfficientNet OR MobileNet | hepatosteatosis OR hepatic steatosis OR hepatic fat OR hepatic lipid OR (liver ADJ3 steatosis) OR (liver ADJ3 fat) OR (liver ADJ3 lipid*) OR (fatty ADJ5 liver) OR ((fatty infiltration) ADJ3 liver) OR fatty hepatosis OR steatohepatosis OR steatohepatitis OR steatopathy OR NAFLD OR non-alcoholic steatohepatitis OR NASH OR Alcoholic liver disease OR Alcohol-related liver disease | ultrasound OR ultra-sound OR sonograph* OR echograph* OR ultrasonograph* OR ultra-sonograph* OR B-mode |

**Table 3:** Subject Heading (MeSH) Terms for OVID MEDLINE ALL database

| **Concept 1** | **Concept 2** | **Concept 3** |
| --- | --- | --- |
| Artificial Intelligence/ or Machine Learning/ or exp Deep Learning/ or exp Neural Networks, Computer/ | exp Fatty Liver/ | Ultrasonography/ |

**Table 4:** Subject Heading (EMTREE) Terms for EMBASE database

| **Concept 1** | **Concept 2** | **Concept 3** |
| --- | --- | --- |
| Artificial Intelligence/ or Machine Learning/ or exp deep learning/ or  exp Artificial Neural Network/ or exp Convolutional Neural Network/ | exp Fatty Liver/ | echography/ |

**Final Search Strategy:**

For each database, we performed
(Keywords: Concept 1 AND Concept 2 AND Concept 3) OR
(Subject Heading Terms: Concept 1 AND Concept 2 AND Concept 3)

**Table 5:** Conflicts in literature review screening and their resolution

| **Citation** | **reviewer1** | **reviewer2** | **consensus** | **consensus_comment** |
| --- | --- | --- | --- | --- |
| Chen J-R, Chao Y-P, Tsai Y-W, Chan H-J, Wan Y-L, Tai D-I, Tsui P-H. Clinical Value of Information Entropy Compared with Deep Learning for Ultrasound Grading of Hepatic Steatosis. Entropy. 2020;22(9):1006. doi:10.3390/e22091006 | Exclude - Raw RF or Quantitative ultrasound parameter inputs | Include | Include | The study compares "US entropy" against a control that uses a CNN-based model. However, for the control group, though the study collects RF data, the input to the CNN model is infact B-mode images. Thus results from the control group are relevant to the systematic review. |
| Ibrahim MN, Blázquez-García R, Lightstone A, Meng F, Bhat M, El Kaffas A, Ukwatta E. Automated Fatty Liver Disease Detection in Point-of-Care Ultrasound B-Mode Images. Journal of Medical Imaging. 2023;10(3):034505. doi:10.1117/1.JMI.10.3.034505 | Exclude - Ground Truth - Not US-based/CT/MRI/Bx | Include | Exclude | Ground Truth - Not US-based/CT/MRI/Bx |
| Tahmasebi A, Wang S, Wessner CE, Vu T, Liu J-B, Forsberg F, et al. Ultrasound-Based Machine Learning Approach for Detection of Nonalcoholic Fatty Liver Disease. Journal of Ultrasound in Medicine. 2023;42(8):1747-1756. doi:10.1002/jum.16194 | Include | Exclude - Unclear whether Model is CNN | Include | Documentation for Google's AutoML (2024) mentions it is based on Google’s leading image recognition approaches including transfer learning and neural architecture search technologies. Highly likely to be based on CNN architecture |
| Zhang P, Huang H, Xiong Q, He X, Liu Y. Feature Analysis and Automatic Classification of B-Mode Ultrasound Images of Fatty Liver. Biomedical Signal Processing and Control. 2023;79:104073.  doi:10.1016/j.bspc.2022.104073 | Exclude - Requisite utcome metrics not reported | Include | Exclude | Requisite outcome metrics not reported |

**APCAPS Ultrasound Dataset: Data Acquisition**

Ultrasound scans were conducted using the Philips – CX50 Portable 2D-Echo Machine. The convex ultrasound probe (C5-1 PureWave Convex) was positioned vertically along the anterior axillary line. Each scan was saved as a brief Digital Imaging and Communications in Medicine (DICOM) format video clip lasting 3-5 seconds, presenting an oblique intercostal view of the right lobe of the liver with 5-6 degrees of angulation.

**APCAPS Ultrasound Dataset: Hepatic Steatosis Consensus Evaluation**

Two radiologists, HF (with 4 years of experience post-speciality training) and SR (with 20 years) performed independent and blinded assessments. Per previously published semi-quantitative criteria,^1^ participants were categorised into one of four HS severity grades (S0 to S3). The severity grades assigned by HF and SR were then simplified into two categories: normal-to-mild HS (S0/S1; with S0 indicating no HS) and moderate-to-severe HS (S2/S3), as reported in previous studies.^1–6^ We chose this categorisation because, compared to mild, moderate-to-severe radiologist-assigned ultrasound HS grades have higher accuracy and reliability,^7^ and stronger associations with cardiometabolic morbidity.^8–10^ In cases of disagreement on these binary labels (n = 50), a third radiologist, AKM (with 8 years) and SR (blinded to their first assessment and to that of AKM) performed further independent reviews. For 28 participants among these 50, there was no consensus between radiologists AKM and SR – these participants were excluded from our gold standard dataset. Ultimately, 219 participants were included in our gold standard dataset.

**Table 6:** Kappa metrics for radiologist grader agreement for hepatic steatosis labels on the APCAPS ultrasound gold standard dataset.

| **Metric** | **Kappa** | **Agreement Level** |
| --- | --- | --- |
| **Initial dataset**  **N = 247 Participants** (Graders HF & SR)**: Inter-reader** |  |  |
| Four grades separately | 0.26 | Fair |
| Binary (S0 vs S1/above) | 0.27 | Fair |
| Binary (S0/S1 vs S2/S3) | 0.44 | Moderate |
| **Subset with disagreements between initial two graders**  **N = 50 Participants** |  |  |
| **Intra-reader** (Grader SR) Binary (S0/S1 vs S2/S3) | -0.24 | Poor* |
| **Inter-reader** (Graders SR & AKM) Binary (S0/S1 vs S2/S3) | 0.14 | Poor |

*the negative value suggests that the agreement is worse than what would be expected by chance alone.

**APCAPS Ultrasound Gold Standard Dataset: Image Processing**

Individual image frames within a given DICOM were cropped and binary thresholded to eliminate ultrasound machine annotations.

**Figure 1:** Image processing for the APCAPS Ultrasound gold standard dataset.


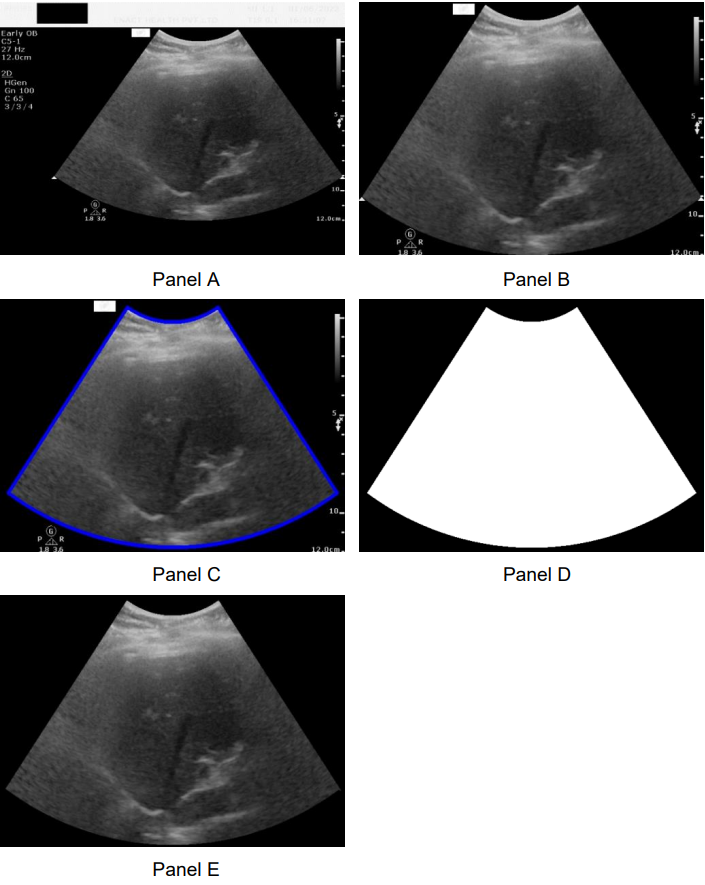
 *Figure with five panels, A, B, C, and D. Panel A: raw image frame before any pre-processing, the black box on the top left was artificially added to redact confidential patient identifiers. Raw image frames in all patient DICOMs were of 600x800 pixel dimensions. Panel B: Image frame after rectangular cropping resulting in an image of 458x680 pixel dimensions (image displayed here is blown up to maintain similar aspect ratios for each panel). Panel C: Showing in blue the contours around the ROI containing pixel data about the liver. Panel D: An image mask created using the coordinates of the contour shown in Panel C. Panel E: Final image used for training, produced through bitwise ‘and’ operation between images shown in panels B and D*

**APCAPS Ultrasound Gold Standard Dataset: CNN Model Development**

We duplicated the pixel arrays of the 1-channel grayscale images, converting them into 3-channel RGB format to match the input requirements of the ImageNet pre-trained InceptionResNetV2 CNN model.^11,12^ We then applied a contrast-limited adaptive histogram equalisation, a popular image enhancement technique particularly effective in medical image processing.^1,13^ We also employed on-the-fly data augmentation involving random combinations of horizontal flips, rotations, translations, and zooming in to increase the volume and diversity of training data.^14^ This process is thought to improve the model’s generalisation performance.^14^ Images were resized, and pixel values were scaled according to the pre-trained model's specifications.^12^ We employed a custom 3 fully connected layer classifier top, with interspersed dropout layers, and a sigmoid activation for the final classification (Figure 2). We explored a grid of values for various hyperparameters, including batch size, optimisers, learning rates, class weightage, and regularisation parameters (label smoothing, dropout values, L1 and L2 values) to determine the optimal configuration. After convergence of the classifier top, we unfroze and fine-tuned the top few layers of the pre-trained model.During training and validation, each image was treated as independent, with the participant DICOM label applied to each constituent image. Similar to previous studies,^1–4,15–19^ this approach served as a form of data augmentation thought to help improve model generalisability. During testing, we calculated the classification probability for each image separately and derived the ensembled probability at the participant level by averaging. Evaluation metrics were calculated using the predicted probability threshold corresponding to the highest Youden index on the receiver operator curve.^20^ Deep learning models were developed using Python (version 3.11.5), Keras (version 2.13.1), and Tensorflow (version 2.13.1) and trained on Standard NC4as T4 v3 (28 GiB memory) GPUs on the Microsoft Azure Platform.

**Figure 2:** Architeture of the model (CNN + neural network classifier top) used for the APCAPS data


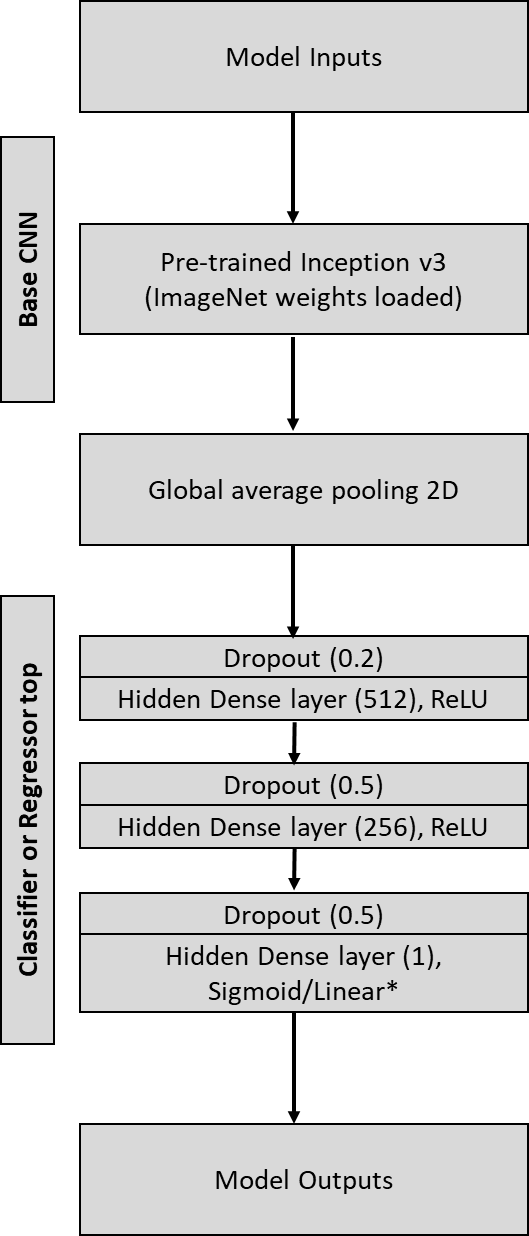


**Methods: Meta-analysis and Evidence Synthesis**For pooling AUCs, we first back-calculated standard errors from reported Confidence Intervals (CIs) and then used a linear random-effects model with logit transformation using R (*meta version 7.0.0)*. We did not include other AUC types (e.g., precision-recall or partial AUCs). Reported AUCs from studies using cross-validation were included as-is, with variation in validation approach (e.g., number of folds) implicitly accounted for in the width of the reported CIs, from which standard errors were derived.

For pooling sensitivities and specificities we first back-calculated participant-level true positives (TP), true negatives (TN), false positives (FP), and false negatives (FN), and then performed bivariate modelling using a linear mixed model with known variances of random effects with logit transformation using R (*mada* version 0.5.11). Where only image-level prevalence was reported and participant-level classification counts were not available, we assumed the image-level prevalence approximated participant-level prevalence for the purposes of back-calculation. Thresholds used to dichotomise predicted probabilities were consistently not reported across studies and was noted as a limitation in the main text. However, we note that the bivariate pooling model is fairly robust to threshold variation, as it jointly models sensitivity and specificity as correlated outcomes. For included studies we calculated the Confidence Intervals (CIs) around sensitivities and specificities, algebraically, using the normal approximation or the Clopper-Pearson exact method, as appropriate.^21^

Heterogeneity measures were calculated using the standard restricted maximum-likelihood estimator, or Holling sample-size adjusted methods, ^22,23^ as appropriate. We qualitatively compared study populations, imaging protocols, reference standards, and CNN architectures to identify plausible sources of heterogeneity; formal subgroup or meta-regression analyses were not feasible because of the small number of studies.

Sensitivity analyses for robustness of synthesised results were not performed due to the small number of studies per outcome. We provide a narrative synthesis of the limitations of the available evidence in the discussion (main text).

**Table 7: Characteristics of included studies that used liver biopsy as ground truth (n = 7)**

| **Ground truth: Liver Biopsy ^a^** | | | | | | | |
| --- | --- | --- | --- | --- | --- | --- | --- |
| **Study / Year** | **Study population**  (Setting/Country, FLD/total,  Steatosis grades, BMI) | **Ultrasound scan protocol & ROIs** | **Total number of images in ground truth dataset before and after augmentation** | **CNN algorithm**  **(Feature extraction + classification) ^b^** | **Validation methods** | **Data**  **Leakage** | **Evaluation metrics (AUC – FLD vs No FLD)** |
| (Brya et al., 2018)^16^ | - Hospital/Poland - 38/55 - Among those with FLD: 52.63% had <=35% steatosis (mild grade) - Overall Population BMI: 45.9 ± 5.6 | - No specific mention of the ROIs in the liver, scanning planes or views. - 10 consecutive images from an image loop sequence from each participant were used – no mention of how this sequence was chosen from all images in the patient’s DICOM. - US images included both the liver and kidney | - 550 (380 FLD, 170 normal) - Did not perform data augmentation | - Pretrained Inception-ResNet-v2 + SVM | - Participant-specific LOOCV producing training and test sets | No | AUC: 0.977 ± 0.021   Sensitivity: 100% Specificity: 88.20% |
| (Zamanian et al., 2021)^17^ |  |  | - 550 (380 FLD, 170 normal) - Normal class images augmented to approximately 380 | Proposed Model: Features extracted from each of the pretrained Inception-ResNet-v2, GoogleNet (Inception v1), AlexNet, and ResNet 101 networks, individually, followed by feature aggregation + SVM. | - 25% of images were used as a hold-out test set, with the remaining used for training | Likely that (original + augmented) images from a single patient were not exclusive to train or test sets. | Proposed model: AUC 0.9999;  Sensitivity: 100%  Specificity: 97.20% |
| (Che et al., 2021)^18^ |  |  | - 550 (380 FLD, 170) - Augmented to 2000 (1000 FLD, 1000 normal) | Images combined with their local phase filtered image and radial symmetry transformed image formed multi-feature inputs to a pretrained multi-scale ResNet + mid-fusion of features + Softmax dense layer | - Used two different paradigms:  (1) Participant-specific LOOCV;  (2) 30% of patients allocated to a hold-out test set: 10 FLD + 5 Non-FLD | No | (1) CV – AUC:  1 (0.99 – 1) [Participant-level]  (2) Hold-out test set:  Sensitivity: 97.2% Specificity: NR |
| (Biswas et al., 2018)^24^ | - Hospital/Portugal - 36/63 - N/R - N/R | - No mention on the scanning planes or views. - One single ROI along the medial axis of the image of the right lobe of the liver from each participant - It appears that US images included both the liver and kidney | - 63 (36 FLD, 27 normal) - Did not perform data augmentation | Custom 22-layer neural net architecture with inception modules + Softmax dense layer | - 10-fold CV | No | AUC: 1  Sensitivity & Specificity : 100% |
| (Chen et al., 2020)^3^** | - Hospital/Taiwan - 126/205 - 38.54% normal, 36.10% mild, 17.07% moderate,  8.29% severe - Overall population BMI: 25.3 ± 3.8 | - 5-independent intercostal scans with manual physician delineated ROIs | - 1025 images - Data augmentation was performed on the training set by random cropping within the original ROIs for the infrequent class to overcome class imbalance (numbers NR) | Pretrained VGG-16 with 3 fully connected layer classifier top with soft max activation. | - Images from 20% of participants formed a hold-out test set, with 5-fold CV in the training set for hyperparameter optimisation. Participants level predictions obtained by majority voting on the images. | No | FLD vs No FLD: AUC 0.71 (0.64 – 0.78), Sensitivity: 73.18%, Specificity: 60%.  (S0 + S1) FLD vs (S2 + S3) FLD:  AUC 0.75 (0.67 – 0.82), Sensitivity: 63.25%, Specificity: 74.82%. |
| (Li et al., 2022)^2^ ^c^ | - Hospital/Taiwan - Development: 370/2899 ; Testing – A : 123/147 ; Testing – B : 68 /112. - Testing – A : 24.49% mild, 23.81% moderate, 35.357% severe; Testing – B : 25.89% mild, 12.5% moderate, 22.32% severe. - Overall population BMI in Testing – A: 26.67, Testing – B: 26.33 | - Images were acquired from four view groups: left liver lobe (longitudinal + transverse), right liver lobe (intercostal), liver-kidney contrast (lower right lobe intercostal + subcostal), and subcostal (with hepatic veins). - In the development cohort, a single patient, had multiple studies, and each study contributed multiple images for algorithm development. - In the testing cohorts, each patient had a single study, with each study having multiple images (across different view groups) | - 200654 images in the development cohort - No mention of data augmentation | ResNet18 (does not mention whether pretrained or not) used to predict a continuous score for each individual image, then ensembled by taking the mean of the image-wise scores within and across each view group for final classification at a given participant’s study level. eff | - Independent hold-out test sets without and with blinding (Testing A & B, respectively) of labels to deep learning development team. | No | FLD vs No FLD:  Testing – A: 0.95 (95% CI 0.91, 0.98) ;  Testing – B: 0.85 (0.77, 0.93)  (S0 + S1) FLD vs (S2 + S3) FLD:  Testing – A:  0.92 (0.88,  0.96)  Testing – B:  0.91 (0.85,  0.97) |
| (Vianna et al., 2023)^4^ | - Hospital/Canada - 142/199 - 43.7% mild, 12.6% moderate, 15.1% severe FLD - Overall population BMI: 30.5 ± 7.8 | - Images were said to be acquired according to the institutional clinical US protocol (not described). - No mention on the scanning planes or views, or ROIs. - A single patient contributed multiple images | - 7529 images (966 normal, 3312 mild, 1683 moderate, and 1568 severe). - Did not perform data augmentation. | - Pretrained VGG-16 architecture + Softmax dense layer - No ensembling was not performed at the patient level, and predictions were obtained on all images in single tests. | - Images from 26% of participants (N = 52, with 12 S0, 17 S1, 11 S2, and 12 S3 grades) were used as a hold-out test set, with the rest for 5-fold cross-validation for hyperparameter optimisation. | No | FLD vs no FLD: AUC 0.85 (95% CI: 0.83, 0.87), Sensitivity: 79%, Specificity: 78%.  (S0 + S1) FLD vs (S2 + S3) FLD : AUC 0.73 (0.71, 0.75), Sensitivity: 76%, Specificity: 58% |

*^a^ all studies except Biswas 2018 employed a cut-off of >5% liver cell steatosis on biopsy for a diagnosis of FLD, while Biswas et al., 2018 did not mention a cut-off; Li et al., 2022 and Vianna et al., 2023 used cut-offs of 5 – 33%, 33 – 66%, >67% for mild, moderate, and severe grades of FLD on biopsy ^b^ when multiple CNN algorithms were studied, only those with the highest AUC are mentioned; algorithms when pre-trained were done so on the ImageNet database; N/A: Not available; LOOCV: Leave one out cross validation. ^c^ Development cohort was graded by visual examination of ultrasounds by radiologists ; Testing A & B, refer to the unblinded and blinded (to deep learning researchers) subsets of histopathology labelled liver ultrasound dataset****;*** *where data augmentation was performed, it was achieved using translation, rotation, flipping, and scaling. **collected RF data but converted to B-mode for the control group model*

**Table 8: Characteristics of included studies that used MRI-PDFF as ground truth (n = 3)**

| **Ground truth: MRI-PDFF values ^a^** | | | | | | | |
| --- | --- | --- | --- | --- | --- | --- | --- |
| **Study / Year** | **Study population**  (Setting/Country, FLD/total,  PDFF %,  BMI) | **Ultrasound scan protocol & ROIs** | **Total number of images in ground truth dataset before and after augmentation** | **CNN algorithm (Feature extraction + classification) ^b^** | **Validation methods** | **Data**  **Leakage** | **Evaluation**  **metrics  (FLD vs No FLD)** |
| (Byra et al.,  2021)^15^ | - Hospital/United States - 118/135 - Among those with FLD, 95% had PDFF ≤30% - Overall population BMI: 31 ± 5 | - Four distinct images per participant were used. - One each from the 3 views in the transverse plane: hepatic veins at the confluence with the inferior vena cava, right portal vein, and right posterior portal vein - One view in the sagittal plane: liver and kidney | - 135 images per view (118 FLD, 17 normal) x 4 views - Images were augmented (appears to be on-the-fly augmentation) | - Pretrained ResNet-50 + Logistic regression (or Lasso Linear regression) for each ultrasound view trained separately. - Followed by, an ensemble model, averaging the outputs of the individual models, was constructed. | - Participant-specific LOOCV producing training and test sets - 4-fold CV for each training set produced. | No | - AUC: 0.91 ± 0.03 - Sensitivity: 0.80 ± 0.05 - Specificity: 0.88 ± 0.05 |
| (Kim et al., 2021)^25^ | - Hospital/South Korea - 39/90 - Mean 11.82% ± 8.74%, and 11.49% ± 5.49% in groups without and with alcohol exposure - N/R | - 2 images per participant were used. - Right intercostal view of the liver - Right intercostal view of the liver containing right renal cortex | - 90 images per view (39 FLD, 51 normal) x 2 views - Each original image was augmented to 39 images. | Features extracted from each of the two views, separately, using pretrained VGG-19, followed by feature concatenation + Sigmoid dense layers | 5-fold CV | No | AUC – 0.87; Sensitivity ~ 70% ; Specificity: 80.5% |
| (Tahmasebi et al., 2023)^19^ | - Outpatient centre/United States - 70/120 - Mean 16.1% ± 0.07% - BMI in FLD: 34.7 ± 7.4, non-FLD: 29.9 ± 7.8 | - Ten distinct images per participant. - Two images from the sagittal-subxiphoid view, 1 from transverse-subxiphoid view, 2 from sagittal-intercostal view, 1 from sagittal-subcostal view, 4 from transverse intercostal view. Different images of the same view were taken at different levels. | - 1191 images (643 FLD + 548 Non-FLD) in the training set and 244 images in the hold-out test set. - No mention of data augmentation. | - Google’s AutoML Vision* - No ensembling was performed at the patient level, and predictions were obtained on all images in single tests. | - Images from 20% of participants (12 FLD + 12 Non-FLD) were used as a hold-out test set. | No | Sensitivity: 72.2% (63.1 – 80.1)  Specificity: 94.6% (88.7 – 98.0) |

*>5% MRI-PDFF values indicated a diagnosis of FLD for Byra et al, and Kim et al.; Tahmasebi used a cut-of >6.4%; ^b^ when multiple CNN algorithms were studied, only those with the highest AUC are mentioned; algorithms when pre-trained were done so on the ImageNet database; N/A: Not available; LOOCV: Leave one out cross validation; *the specific implementations of the underlying model architecture is proprietary to Google and not disclosed; however, the documentation mentions it is based on Google’s leading image recognition approaches including transfer learning and neural architecture search technologies – thus highly likely to be based on convolutional neural network architectures*

**Table 9: Characteristics of included studies that used B-mode ultrasound graded by radiologists as ground truth (n = 7)**

| **Ground truth: Ultrasound grading by radiologists ^a^** | | | | | | | |
| --- | --- | --- | --- | --- | --- | --- | --- |
| **Study / Year** | **Study population**  (Setting/Country, FLD/total,  Steatosis grades, BMI) | **Ultrasound scan protocol & ROIs** | **Total number of images in ground truth dataset before and after augmentation** | **Key Preprocessing,  CNN algorithm (Feature extraction + classification)^b^** | **Validation methods** | **Data**  **Leakage** | **Evaluation metrics (AUC)^c^** |
| (Cao et al., 2019)^5^ | - Hospital/China - 134/240 - 44.16% had no FLD, 23.75% had mild, 27.91% moderate, 4.17% severe FLD. - N/R | - The ROI was a (single) clinician-selected avascular and lesion-free site, at a 5 cm subcutaneous depth of the standard section of the right liver. | - 852 images (no description of the study population from whom these images were taken, or of the proportions of FLD and normal) - 2582 images** | 3-convolutional layers + 2-layer FC neural net with Softmax dense layer | Images from the 240 participants  described in the  study population  formed the hold-out test set. | Cannot  Comment | FLD vs no FLD: 0.933  (S0 + S1) FLD vs (S2 + S3) FLD:  0.958 |
| (Chou et al., 2021)^1^ | - Hospital/Taiwan - xx/2070 - N/R - N/R | - No mention of the specific ROI in the liver, scanning planes or views. Appears that US images used show both liver and kidney. - Multiple images (not clear how many) per patient were used. | - 21855 images (11307 normal, mild FLD 4467, moderate 3155, severe 2926) - Images were augmented (appears to be on-the-fly augmentation) | - Pretrained ResNet-50 v2 + Softmax dense layer - No ensembling was performed at the patient level, and predictions were obtained on all images in single tests | Images from 20% of participants (N = 418, ) were used as a hold-out test set, with the rest for training. | No | FLD vs no FLD: 0.985; Sensitivity: 91.8%; Specificity: NR  (S0 + S1) FLD vs (S2 + S3) FLD: 0.996; Specificity: 98.8%; Sensitivity: NR |
| (Constantinescu et al., 2021)^26^ | - Outpatient clinic/Romania - 30/60 - N/R - N/R | - No mention of the specific ROI in the liver, scanning planes or views. Appears that US images used show both liver and kidney - Multiple non-overlapping crops or patches from a single patient US image frame were used. | - Number of original images not mentioned - 629 images (332 FLD, 297 normal) | Pretrained Inception v3 + Sigmoid dense layer | Approximately 20% of the images (N = 133 with 70 FLD) in the dataset formed a hold-out test set | Likely that image patches from a single patient were not exclusive to either train or test sets. | FLD vs no FLD: 0.93 ; Sensitivity: 88.9% ; Specificity: NR |
| (Rhyou et al., 2021)^27^ | Used two study populations:  1. Byra et al., 2018 study population – Refer Table XX  2. Hospital/South Korea (Samsung Medical database); N/R; N/R; N/R | - Byra 2018, details – Refer Table X - Images in Samsung database were of parasagittal section of liver. Cannot comment whether one participant contributed one or more images as study population details are N/R - Images from both populations included both liver and kidney | - Byra: 550 images (380 FLD, 170 normal); Samsung: 2650 images (1150 FLD, 1500 normal) - Images were augmented (appears to be on-the-fly augmentation) | Semantic segmentation of the liver and kidney from the images inputted into a pre-trained Inception v3 + Softmax dense layer | 60% of the total images were used for training, with 20% for validation, and the remaining 20% forming the held-out test set. | Cannot comment on the Samsung database;  Yes for when using Byra et al. | Multi-class normal vs mild vs moderate vs severe FL – AUC: 1, sensitivity & specificity : 100% (when trained on both datasets with testing on the Byra et al., 2018 dataset) |
| (Huang et al., 2023)^28^ | Used two study populations:  1. Byra et al., 2018 study population – Refer Table XX  2. Hospital/China, N/R, N/R, N/R. | - Refer Table X for details on Byra et. al., dataset - No mention of the specific scanning planes or views. - Two ROIs (one near field, and one far field) per ultrasound image was extracted with the help of clinicians. - Unclear if a single patient contributed more than one image. | - 2416 images (588 normal, 659 mild, 692 moderate, 477 severe) - No mention of data augmentation | - EfficientNet-B3 (not pretrained) with adaptive coordination attention + fully connected layer. | 10-fold cross validation | Cannot comment | Multi-class normal vs mild vs moderate vs severe FLD, micro/macro average AUC: 0.96 (Huang’s dataset), Sensitivity: 0.969;  FLD vs No FLD on Byra dataset: AUC: 0.989 |
| (Yang et al., 2023)^6^ | - Community/China - 615/928 - 48% mild, 7.3% moderate, 11% severe. - Overall Population: 23.8 ± 3.2 | - Two images per participant were concatenated and used – epigastric longitudinal scanning in the median sagittal plane in the subxiphoid region + right subcostal scanning along the right subcostal margin. | - 928 (two images from each participant were concatenated into one) - No mention of data augmentation | Custom 2-section Neural Network with 3 ResNet inspired blocks to extract image features and predict ‘bright liver’, ‘intra-hepatic duct blurring’, ‘impaired diaphragm visualization’, which were then concatenated and passed into a fully connected layer for classification. | A hold-out test set of 186 (20%) of participants. | No | FLD vs no FLD: 0.90; Sensitivity: 88.6% ; Specificity: 90.5%.  (S0 + S1) FLD vs (S2 + S3) FLD : 0.84; Sensitivity: 76.% ; Specificity: 92.8%. |
| (Zhu et al., 2022)^29^ | - Hospital/China - 12/16 - 25% each of normal, mild, moderate, and severe FLD - N/R | - No mentions of the specific ROI in the liver, scanning planes, or views. - Each patient contributed two images. | - 32 images (8 each in normal, mild, moderate, and severe FLD classes respectively) - Using a moving window small (original) image patches were extracted, and further augmented using a pixel-based differential image data extension strategy to create 3000 samples. | A shallow 10-layer custom CNN with skip connections and a four node softmax dense layer. | 50% of the original image patches (1000) were used as a hold-out testing set. | Likely that image patches from a single patient were not exclusive to either train or test sets. | Multi-class sensitivity*: 83%, specificity*: 95%. |

*^a^ Chou et al 2021, used the same criteria for FLD grading as the APCAPS protocol; Cao et al 2019, used the FLD grading criteria as previously described^30^ ;Constantinescu et al., 2021 diagnosed FLD by the presence of diffuse hyperechoic structure, deep beam attenuation, and increased liver echogenicity in relation to the right kidney; Yang et al., 2023 graded based on bright liver, intrahepatic duct blurring, and impaired visualization of more than half of the diaphragm; Rhyou et al., 2021 and Huang et al., 2023 did not provide the criteria used for FLD grading; ^b^when multiple CNN algorithms were studied, only those with the highest AUC are mentioned; algorithms when pre-trained were done so on the ImageNet database; ^c^ AUROC of 1 for Rhyou et. al., was calculated from sensitivity and specificity values of 1; N/A : Not available *no mention of whether this is a micro/macro/weighted average*  ;; ** Did not mention how they were augmented

**Table 10**: **Ultrasound scanner models used across different studies**

| **Study** | **Ultrasound Scanner/s Used** |
| --- | --- |
| (Brya et al., 2018)^16^ | GE Vivid E9 US |
| (Zamanian et al., 2021)^17^ |  |
| (Che et al., 2021)^18^ |  |
| (Biswas et al., 2018)^24^ | CX_C50 (Philips) |
| (Chen et al., 2020)^3^ | Model 3000, Terason |
| (Li et al., 2022)^2^ | ATL HDI 5000, GE Healthcare LOGIQ E9, GE Healthcare LOGIQ S8, Aloka SSD 5500, Hitachi VISION Avius, Hitachi VISION Preirus, Philips EPIQ 7G, Philips HD15, Philips iU22, Siemens S2000, SuperSonic Imagine Aixplorer, Toshiba TUS-A300, Toshiba Xario |
| (Vianna et al., 2023)^4^ | iU22 (Philips), Aplio 500 and i800 (Canon Medical Systems), Acuson S2000 and S3000, (Siemens Healthineers) Sequoia (Siemens Healthineers), and LOGIQ E9 (GE HealthCare) |
| (Byra et al., 2021)^15^ | Siemens S3000 |
| (Kim et al., 2021)^25^ | PHILIPS or GE scanners (C5-1/ABD, PHILIPS; LOGIQ E10, GE) |
| (Tahmasebi et al., 2023)^19^ | LOGIQ E10 (GE) |
| (Cao et al., 2019)^5^ | Mindray Resona 7 color US |
| (Rhyou et al., 2021)^27^ | Siemens Acuson Sequoia |

*Studies that did not report ultrasound scanner models are not included in this table.*

**Figure 3:** Simple schematic representation of internal vs external validation

**Internal Hold-Out Validation:**

Validation set: for hyperparameter optimisation

(Hold-out) Test set: for reporting evaluation metrics

Training set: for learning model parameters

A single dataset is partitioned into three exclusive datasets – training, validation, and testing. Sometimes the validation set is omitted (in this case the hyperparameter optimisation may be done using the training set).

**Internal Cross-Validation:**

In k-fold cross-validation, the single dataset is split into k equal parts:

- The model is trained on k-1 parts and tested on the remaining part.
- This process is repeated k times, and the evaluation metrics on the “testing” parts are averaged.

**External Hold-Out Validation:**

Dataset 1 used for internal-hold-out, or internal-cross-validation

A distinct second dataset, used for reporting evaluation metrics of the model trained on dataset 1.

**Data Leakage:**

AUCs for the any severity HS detection task, for the studies where we couldn’t definitely exclude the possibility of data leakage, ranged from 0.93 – 0.999, which was higher than that for the low-risk of bias studies included in the pooling of AUCs for the same task, 0.71 – 0.995 (see main text).

**Table 11:** Studies (n=6) with possible data leakage between training/test sets (or folds) along with their reported evaluation metrics

| **Sr** | **Study** | **Reported Evaluation Metrics** | | |
| --- | --- | --- | --- | --- |
|  |  | **Any Severity HS detection:** (S1 or higher) vs (S0) | **Moderate-to-severe HS detection:** (S2/S3) vs (S1/S0) | **Multi-class HS detection** |
| **1** | (Zamanian et al., 2021)^17^ | AUC: 0.999;  Sensitivity: 100%, Specificity: 97.2% | NR | NR |
| **2** | (Cao et al., 2019)^5^ | AUC: 0.933 | AUC: 0.958 | NR |
| **3** | (Constantinescu et al., 2021)^26^ | AUC: 0.93  Sensitivity: 88.9% | NR | NR |
| **4** | (Rhyou et al., 2021)^27^ | NR | NR | 1 |
| **5** | (Zhu et al., 2022)^29^ | NR | NR | Sensitivity: 83%,  Specificity: 95% |
| **6** | (Huang et al., 2023)^28^ | On the Byra et. al., 2018 dataset – AUC: 0.989 | NR | AUC: 0.96 |

AUC: area under the receiver operator curve, NR: Not reported

**Research question:** Diagnostic accuracy of convolutional neural networks in detecting steatosis from conventional B-mode ultrasound images: A systematic review and meta-analysis (AJ & CA – blinded independent review + consensus)

**Table 12:** Risk of bias and applicability assessment

Of the 17 included articles in the review, we excluded 8 studies due to concerns of bias or applicability because of possible data leakage inflating index test performance metrics,^5,17,26–29^ insufficient reporting on index tests,^1^ reference standards,^24,27–29^ patient selection,^5,27,28^ or the flow and timing domains.^24^

| **Reference test:** Liver biopsy | | | | | | | | |
| --- | --- | --- | --- | --- | --- | --- | --- | --- |
| **Study reference/**  **year** | **Risk of bias** | | | | **Applicability concerns** | | | **Comments** |
|  | **Patient selection** | **Index Test** | **Reference Standard** | **Flow and timing** | **Patient selection** | **Index Test** | **Reference Standard** |  |
| (Brya et al., 2018)^16^ | LR | LR | LR | LR | LR | LR | LR | **Index Test reporting – (S1 or higher) vs (S0) HS:** CIs or SEs for AUC not reported (mean±sd over cv folds are reported), so cannot be included in the pooling of AUC. But study can be included in the quantitative synthesis of sensitivity /specificity (which are reported). **(S2/S3) vs (S1/S0) HS:** No metrics reported. |
| (Zamanian et al., 2021)^17^ | LR | **UR** | LR | LR | LR | LR | LR | Unable to exclude the possibility of data leakage between (train-test dataset) |
| (Che et al., 2021)^18^ | LR | LR | LR | LR | LR | LR | LR | **Index Test reporting – (S1 or higher) vs (S0) HS**: Specificity is not reported, so cannot be included in the pooling of sensitivity/specificity. AUC (CI) reported can be used for pooling of AUC. **(S2/S3) vs (S1/S0) HS:** No metrics not reported. |
| (Biswas et al., 2018)^24^ | LR | LR | **UR** | **UR** | LR | LR | LR | No cut-off for reference standard is reported.  No time difference between index test and reference standard is reported |
| (Chen et al., 2020)^3^ | LR | LR | LR | LR | LR | LR | LR |  |
| (Li et al., 2022)^2^ | LR | LR | LR | LR | LR | LR | LR | For both classification tasks, study does not report sensitivity & specificity so cannot be used for bivariate pooling of these metrics. But reported AUC (CI) can be used for pooling of AUC metric. |
| (Vianna et al., 2023)^4^ | LR | LR | LR | LR | LR | LR | LR | Time diff between index test and reference standard is 1 year (acceptable as NAFLD has a chronic course) |
| **Reference test:** MRI-PDFF | | | | | | | | |
| (Byra et al., 2021)^15^ | LR | LR | LR | LR | LR | LR | LR | **Index Test reporting – (S1 or higher) vs (S0) HS:** CIs or SEs for AUC not reported (mean±sd over cv folds are reported), so cannot be included in the pooling of AUC. But study can be included in the quantitative synthesis of sensitivity /specificity (which are reported). **(S2/S3) vs (S1/S0) HS:** No metrics reported. |
| (Kim et al., 2021)^25^ | LR | LR | LR | LR | LR | LR | LR | **Index Test reporting – (S1 or higher) vs (S0) HS:** CIs or SEs for AUC not reported (only mean over cv folds are reported), so cannot be included in the pooling of AUC. But study can be included in the quantitative synthesis of sensitivity /specificity (which are reported). **(S2/S3) vs (S1/S0) HS:** No metrics reported. |
| (Tahmasebi et al., 2023)^19^ | LR | LR | LR | LR | LR | LR | LR | **Index Test reporting – (S1 or higher) vs (S0) HS:** AUC not reported. But study can be included in the quantitative synthesis of sensitivity /specificity (which are reported).**(S2/S3) vs (S1/S0) HS:** No metrics reported. |
| **Reference test:** Radiologist evaluation of B-mode ultrasound* | | | | | | | | |
| (Cao et al., 2019)^5^ | **UR** | **UR** | LR | LR | LR | LR | LR | Not enough information was provided to exclude dataleakage between train/test splits.  No description of train set population (or its provenance) |
| (Chou et al., 2021)^1^ | LR | LR | LR | LR | LR | **HR** | LR | **Index Test reporting –** For both binary classification tasks, **(S1 or higher) vs (S0) HS**: Confidence Intervals (or standard errors) for AUC are not reported. Specificity is not reported. **(S2/S3) vs (S1/S0) HS**: Confidence Intervals (or standard errors) for AUC is not reported. Sensitivity is not reported.  There are concerns that the results of the index test differ from the review question and thus may not be applicable to the quantitative synthesis. |
| (Constantinescu et al., 2021)^26^ | LR | **HR** | LR | LR | LR | LR | LR | Data leakage between image patches in train/test sets highly likely |
| (Rhyou et al., 2021)^27^ | **UR for Samsung**  LR for Byra | **- HR for Samsung dataset**  - LR for Byra et al. 2018, dataset | **- UR for Samsung dataset**  - LR for Byra et al. 2018, dataset. | LR | LR | LR | LR | Not enough information was provided  (1) to exclude data leakage between train/test splits  (2) regarding the radiological criteria for grading  No description of the population |
| (Huang et al., 2023)^28^ | **UR** | **- UR for Huang**  - LR for Byra et al. | **- UR for Huang**  - LR for Byra et al. | LR | LR | LR | LR | No description of population or reference standard, or train/test splitting procedures, thus unable to exclude the possibility of data leakage. |
| (Yang et al., 2023)^6^ | LR | LR | LR | LR | LR | LR | LR | For both classification tasks, study does not report AUC (CI) or (SE) so cannot be used for pooling AUC metric. But reported sensitivity and specificity can be used for bivariate modelling and pooling of these metrics. |
| (Zhu et al., 2022)^29^ | LR | **HR** | **UR** | LR | LR | LR | LR | Criteria for reference standard not mentioned  Data leakage between train/test likely |

*Flow and timing domain for studies using radiologist-assigned HS grades are irrelevant and thus all such studies are designated as LR for this domain;
LR – Low Risk, HR – High Risk, UR – Unknown Risk, AUC – area under the receiver operator curve, CI – confidence intervals, SE – standard errors, HS – hepatic steatosis; S0, S1, S2, S3 refers to normal (or no HS), mild, moderate, or severe HS respectively.

**Meta-Analysis: Quantitative Synthesis of Sensitivity & Specificity for identifying any severity HS**

Among the ten considered studies, seven studies (conducted on distinct datasets) reported sensitivity and specificity for any severity HS identification task, (S1 or higher) vs (S0), including 583 (unseen) participants. Among them, three studies utilised histological gold standard, three others used MRI-PDFF, and one utilised a radiologist-assigned US HS grade. The weighted prevalence of gold standard-defined any severity HS (S1 or higher) across studies was 64.83% (n=378).

For back-calculation of participant-level true positives (TP), true negatives (TN), false positives (FP), false negatives (FN) from reported sensitivity, specificity, and prevalences we made the following assumptions: (1) In two studies^3,25^ the any severity HS (S1 or higher) prevalences reported pertained to the whole dataset, and not the hold-out or cross-validated sets on which sensitivity and specificity evaluation metrics were reported; given random data splits, we assumed the prevalences in the whole dataset and its respective evaluation set would be similar, (2) In two other studies^4,19^ only image-level measures for sensitivity and specificity were reported; we assumed that the reported image-level metrics would be fairly similar to that expected at participant level.

Pooled sensitivities and specificities obtained from bivariate diagnostic modelling revealed strong discrimination between target classes, 79.30% (71.70 – 85.30) and 81.20% (71.40 – 88.20), respectively. We do not report the area under this summary receiver operator curve (sAUC), as it has been previously shown to be a biased estimate for pooling AUCs^31^ – instead we provide the direct meta-analysis of back-calculated standard errors from study-reported CIs around AUCs (see main text).

**Figure 4** (Panels A & B)**:** Calculated participant-level sensitivities and specificities of the studies (from the back-calculated participant-level true positives, true negatives, false positives, false negatives from reported sensitivities, specificities, and prevalences) included in the meta-analysis (n = 7).

**Panel A:** Forest Plots


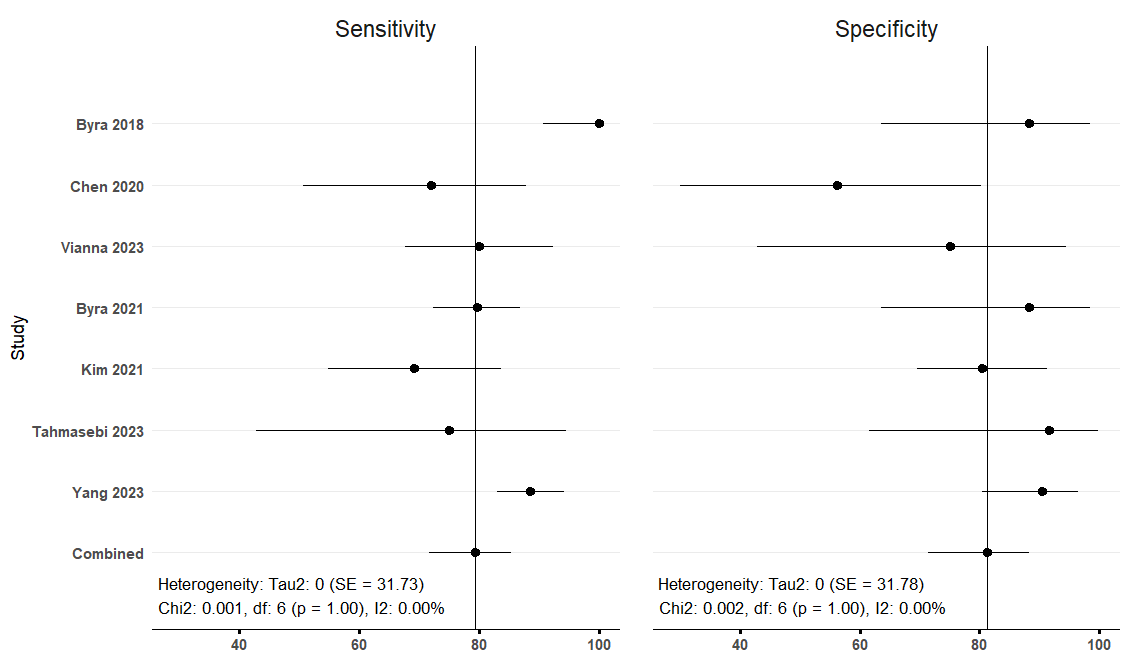


*Heterogeneity metrics reported in the figure are Hunter-Schmidt estimator with a small sample-size correction in univariate modelling of sensitivity and specificity (separately). Holling sample-size adjusted and unadjusted bivariate heterogeneity measures were 2.90 – 6.70%, and 51.30 – 73.30%, respectively.*

**Panel B:** Sensitivity vs (100 – Specificity) with confidence intervals denoted by error bars

**
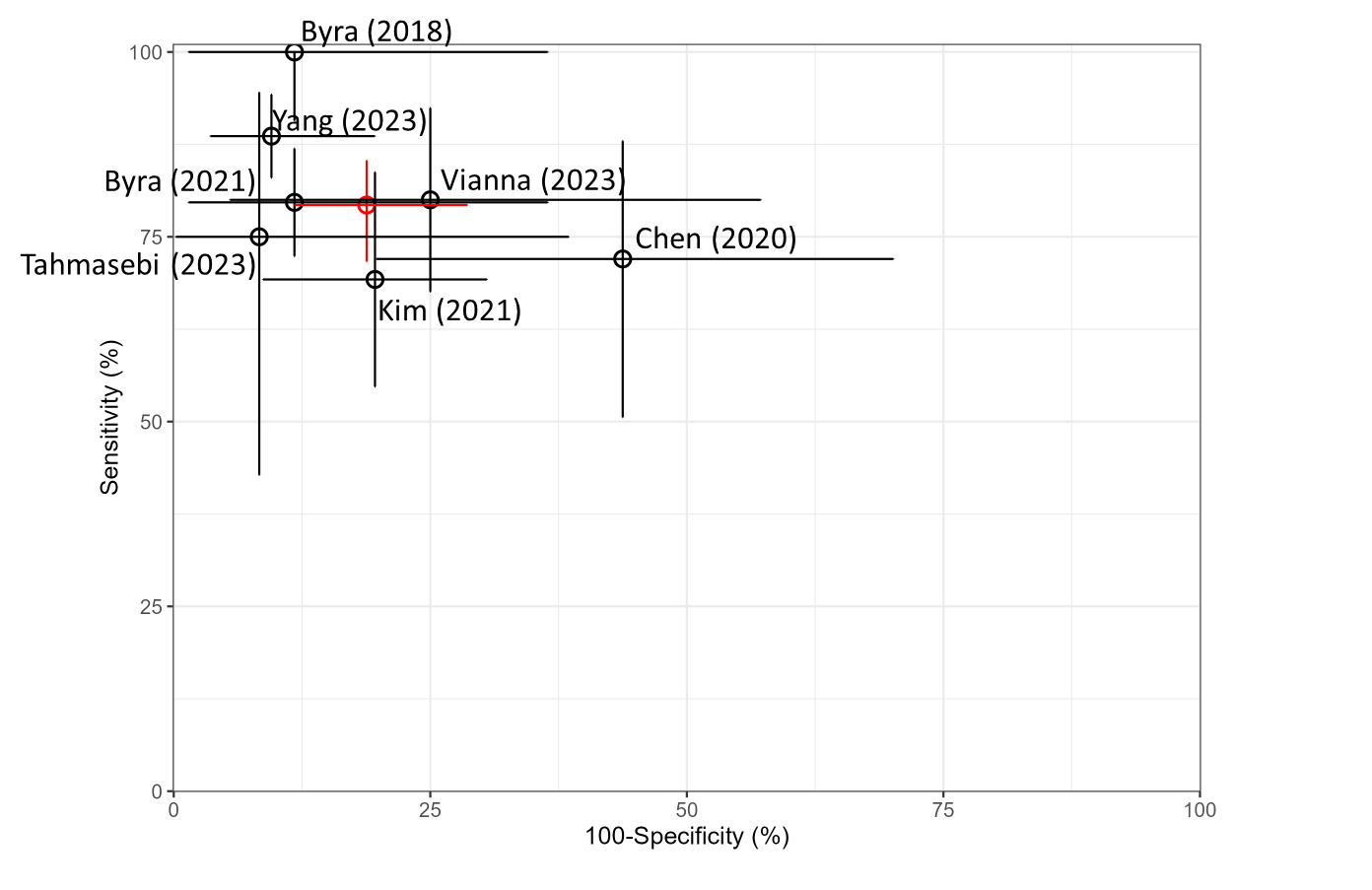
**

*The pooled sensitivity and specificity is shown in red.*

**Meta-Analysis: Quantitative Synthesis of Sensitivity & Specificity for identifying any moderate-to-severe HS**

Only three^3,4,6^ of the 10 studies considered for quantitative synthesis reported sensitivities and specificities for this (S2/S3) vs (S1/S0) HS task. This did not meet the pre-defined criterion of being reported in at least 5 unique datasets thus we could not quantitative pool sensitivity and specificity for the moderate-to-severe HS identification task.

**Table 13: Comparison of Overall and Subsample Participant Characteristics**

| **Characteristic*** | **Overall Sample APCAPs  2022-23 followup**  **(*N* = 2057)** | **Sample selected for radiological analysis**  **(*N* = 261)** | **Sample with ultrasound gold standard labels**  **(*N* = 219)** | ***p values***  *(for tests comparing overall sample, N = 2057 and gold standard subset, N = 219)* |
| --- | --- | --- | --- | --- |
| **Age (mean** ± **SD)** | 58.42 ± 6.96 | 58.80 ± 6.70 | 58.85 ± 6.69 | 0.38 |
| **Sex** (Female) | 1233 (59.94%) | 163 (62.45%) | 135 (61.64%) | 0.62 |
| **Body Mass Index-Categories**** | | | | |
| Underweight (<18.5) | 262 (12.74%) | 37 (14.74%) | 33 (15.57%) | 0.15 |
| Normal (18.5 – 22.9) | 782 (38.02%) | 103 (41.04%) | 90 (42.45%) |  |
| Overweight (23 – 27.4) | 682 (33.16%) | 77 (30.68%) | 65 (30.66%) |  |
| Obese (≥27.5) | 331 (16.09%) | 34 (13.55%) | 24 (11.32%) |  |

*****no missing values, ******cut-offs as per the WHO criteria for body mass index for Asians

**Variation in evaluation metrics when reported ensembled at participant-level vs that at the image level**

| **Metrics Level** | **Participant-level**  (By averaging prediction probabilities across all images per participant) | **Image-level** |
| --- | --- | --- |
|  | (*N* = 66 participants) | (*N* = 4001 images, from 66 participants) |
| **AUROC** | 0.90 (0.77, 1.00) | 0.84 (0.82, 0.86) |
| **Sensitivity** | 80.00 (51.91, 95.67) | 74.92 (72.06, 77.63) |
| **Specificity** | 98.03 (89.55, 99.95) | 84.85 (83.52, 86.11) |

**Table 14:** Evaluation metrics of the APCAPS trained model reported on the APCAPS test set, at participant and image levels

These variations noted above were likely due to observed variations in prediction probabilities (as demonstrated by the standard deviation) for the target class across the series of images in a given participant’s DICOM.

**Figure 5:** Histogram showing distribution of standard deviation of prediction probabilities (across images) per participant

**
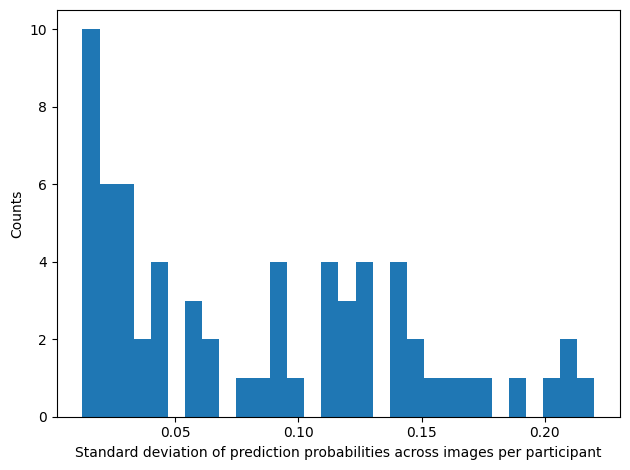
**

**Figure 6: Funnel Plots for pooling area under receiver operator curve outcomes**

**
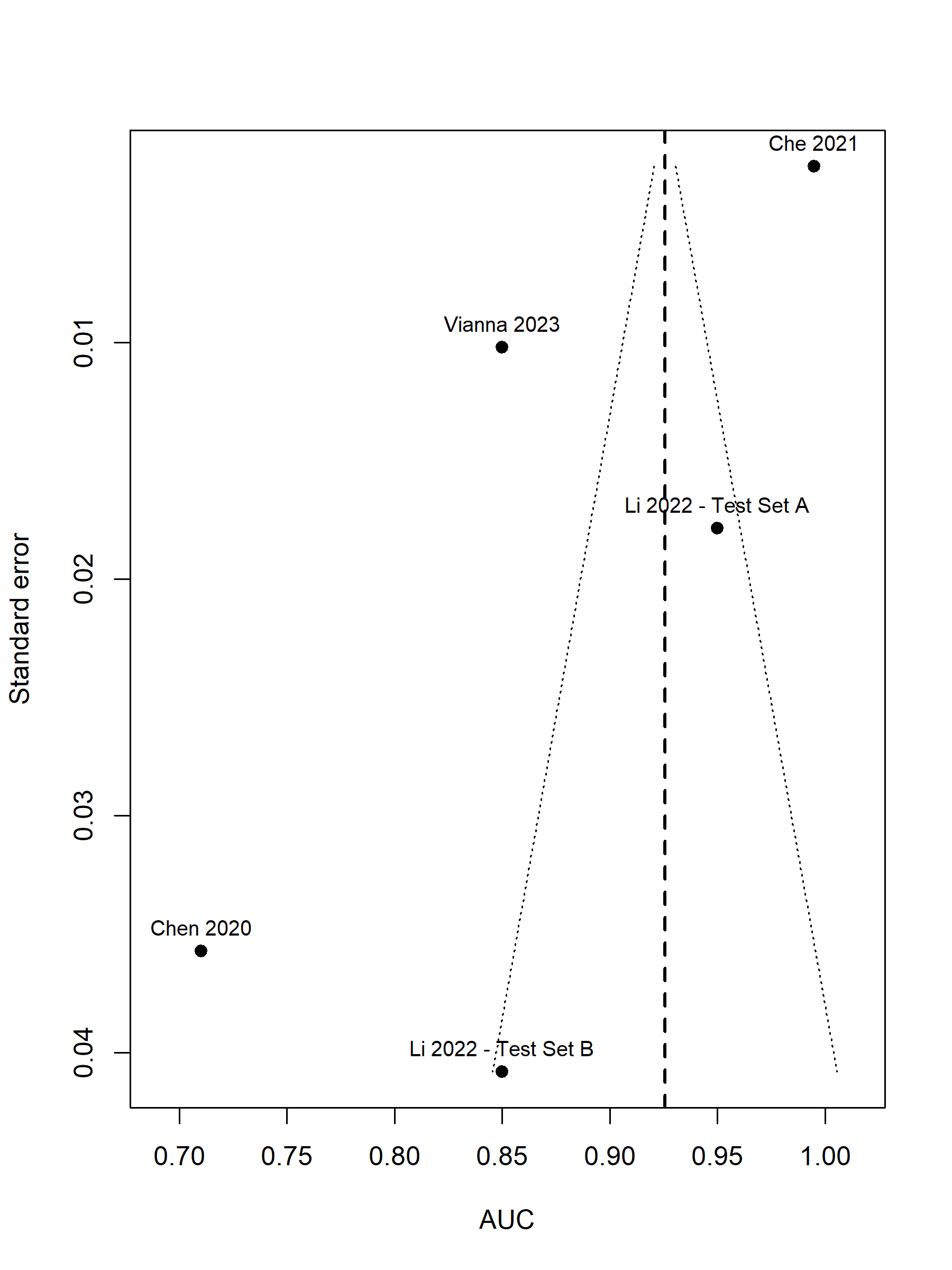

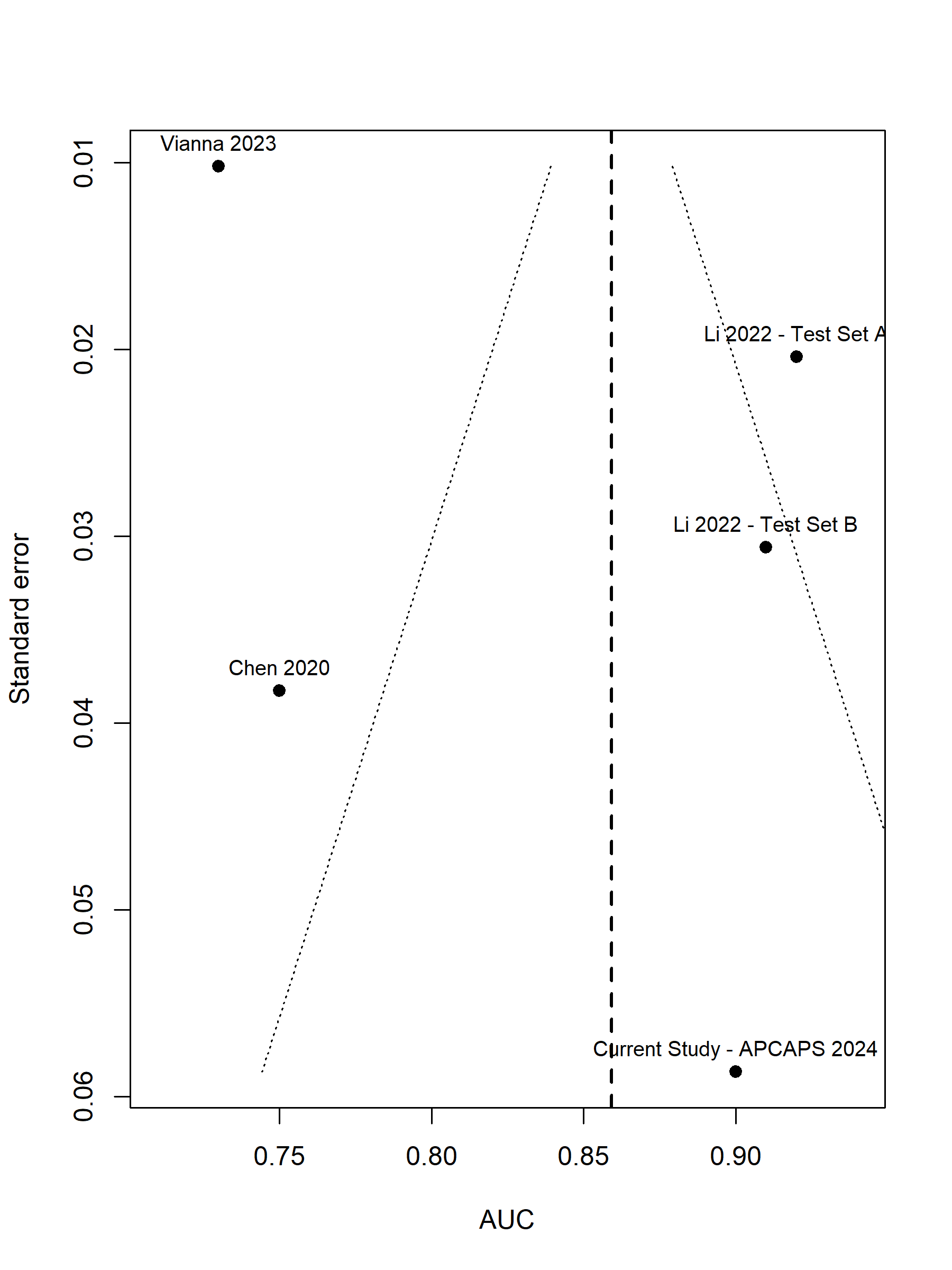
***The left panel shows funnel plot for the AUC (Area under receiver operator curve) pooling for the any severity HS (S0 vs. S1 or above) identification task, and the right panel shows that for moderate-to-severe HS (S2/S3 vs. S0/S1) identification task. Each point represents an individual study. The dashed vertical line represents the overall pooled AUC estimate from the random-effects meta-analysis. The dotted lines indicate the 95% pseudo-confidence limits, defining the expected funnel shape in the absence of publication bias or study heterogeneity.*

**Figure 7: Deek’s funnel plot for Diagnostic Odds Ratio** (for the bivariate pooling of sensitivity and specificity outcomes from studies reporting for the any severity HS identification task)

*
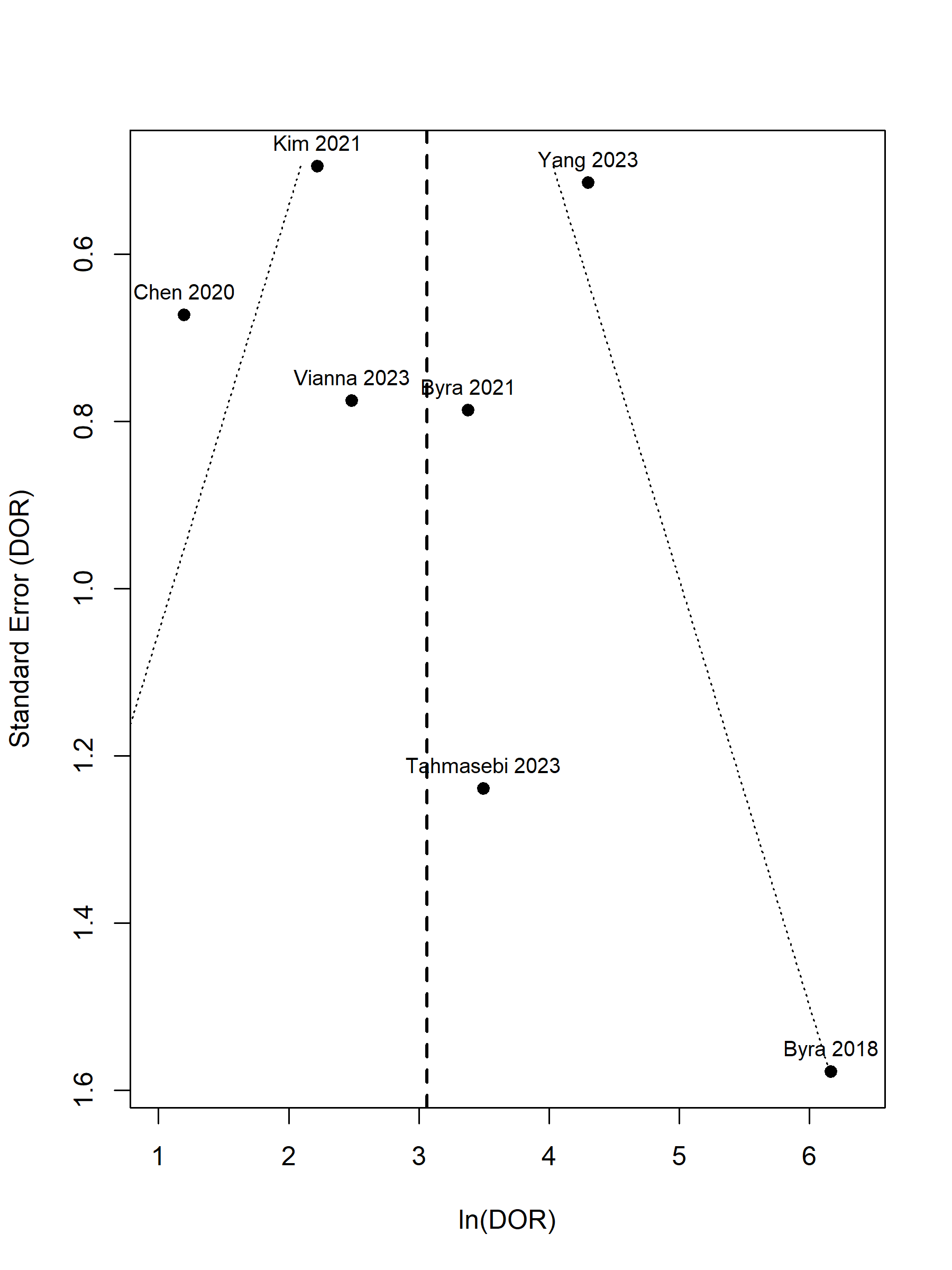
*

*DOR: Diagnostic Odds’ ratio. Each point represents an individual study. The dashed vertical line represents the overall pooled lnDOR estimate from the meta-analysis (we report bivariate pooled sensitivity/specificity in the main text). The dotted lines indicate the 95% pseudo-confidence limits, defining the expected funnel shape in the absence of publication bias or study heterogeneity.*

**References (for supplementary information):**

1 Chou TH, Yeh HJ, Chang CC, *et al.* Deep learning for abdominal ultrasound: A computer-aided diagnostic system for the severity of fatty liver. *Journal of the Chinese Medical Association* 2021; **84**: 842–50.

2 Li B, Yan K, Le L, *et al.* Accurate and generalizable quantitative scoring of liver steatosis from ultrasound images via scalable deep learning. *World J Gastroenterol* 2022; **28**: 2494–508.

3 Chen JR, Chao YP, Tsai YW, *et al.* Clinical value of information entropy compared with deep learning for ultrasound grading of hepatic steatosis. *Entropy* 2020; **22**. DOI:10.3390/e22091006.

4 Vianna P, Calce SI, Boustros P, *et al.* Comparison of Radiologists and Deep Learning for US Grading of Hepatic Steatosis. *Radiology* 2023; **309**. DOI:10.1148/radiol.230659.

5 Cao W, An X, Cong L, Lyu C, Zhou Q, Guo R. Application of Deep Learning in Quantitative Analysis of 2-Dimensional Ultrasound Imaging of Nonalcoholic Fatty Liver Disease. *Journal of Ultrasound in Medicine* 2020; **39**: 51–9.

6 Yang Y, Liu J, Sun C, *et al.* Nonalcoholic fatty liver disease (NAFLD) detection and deep learning in a Chinese community-based population. *Eur Radiol* 2023; **33**: 5894–906.

7 Hernaez R, Lazo M, Bonekamp S, *et al.* Diagnostic Accuracy and Reliability of Ultrasonography for the Detection of Fatty Liver: A Meta-Analysis. *Hepatology* 2011; **54**: 1082.

8 Song Q, Ling Q, Fan L, *et al.* Severity of non-Alcoholic fatty liver disease is a risk factor for developing hypertension from prehypertension. *Chin Med J (Engl)* 2023; **136**: 1591–7.

9 Song Q, Liu S, Ling QH, *et al.* Severity of Nonalcoholic Fatty Liver Disease is Associated With Cardiovascular Outcomes in Patients With Prehypertension or Hypertension: A Community–Based Cohort Study. *Front Endocrinol (Lausanne)* 2022; **13**. DOI:10.3389/fendo.2022.942647.

10 Han JM, Cho JH, Kim HI, *et al.* Greater Severity of Steatosis Is Associated with a Higher Risk of Incident Diabetes: A Retrospective Longitudinal Study. *Endocrinology and Metabolism* 2023; **38**: 418–25.

11 Mishkin D, Sergievskiy N, Matas J. Systematic evaluation of CNN advances on the ImageNet. 2016; published online June 7. DOI:10.1016/j.cviu.2017.05.007.

12 Keras Applications. https://keras.io/api/applications/ (accessed March 13, 2024).

13 Zaridis DI, Mylona E, Tachos N, *et al.* Region-adaptive magnetic resonance image enhancement for improving CNN-based segmentation of the prostate and prostatic zones. *Scientific Reports 2023 13:1* 2023; **13**: 1–14.

14 Mumuni A, Mumuni F. Data augmentation: A comprehensive survey of modern approaches. *Array* 2022; **16**: 100258.

15 Byra M, Han A, Boehringer AS, *et al.* Liver Fat Assessment in Multiview Sonography Using Transfer Learning With Convolutional Neural Networks. *Journal of Ultrasound in Medicine* 2022; **41**: 175–84.

16 Byra M, Styczynski G, Szmigielski C, *et al.* Transfer learning with deep convolutional neural network for liver steatosis assessment in ultrasound images. *Int J Comput Assist Radiol Surg* 2018; **13**: 1895–903.

17 Zamanian H, Mostaar A, Azadeh P, Ahmadi M. Implementation of combinational deep learning algorithm for non-alcoholic fatty liver classification in ultrasound images. *J Biomed Phys Eng* 2021; **11**: 73–84.

18 Che H, Brown LG, Foran DJ, Nosher JL, Hacihaliloglu I. Liver disease classification from ultrasound using multi-scale CNN. *Int J Comput Assist Radiol Surg* 2021; **16**: 1537–48.

19 Tahmasebi A, Wang S, Wessner CE, *et al.* Ultrasound-Based Machine Learning Approach for Detection of Nonalcoholic Fatty Liver Disease. *Journal of Ultrasound in Medicine* 2023; **42**: 1747–56.

20 Smits N. A note on Youden’s J and its cost ratio. *BMC Med Res Methodol* 2010; **10**: 1–4.

21 Ying GS, Maguire MG, Glynn RJ, Rosner B. Calculating Sensitivity, Specificity, and Predictive Values for Correlated Eye Data. *Invest Ophthalmol Vis Sci* 2020; **61**. DOI:10.1167/IOVS.61.11.29.

22 Viechtbauer W. Bias and Efficiency of Meta-Analytic Variance Estimators in the Random-Effects Model. *J Educ Behav Stat* 2005; **30**: 261–93.

23 Holling H, Böhning W, Masoudi E, Böhning D, Sangnawakij P. Evaluation of a new version of I2 with emphasis on diagnostic problems. *Commun Stat Simul Comput* 2020; **49**: 942–72.

24 Biswas M, Kuppili V, Edla DR, *et al.* Symtosis: A liver ultrasound tissue characterization and risk stratification in optimized deep learning paradigm. *Comput Methods Programs Biomed* 2018; **155**: 165–77.

25 Kim T, Lee DH, Park EK, Choi S. Deep learning techniques for fatty liver using multi-view ultrasound images scanned by different scanners:development and validation study. *JMIR Med Inform* 2021; **9**. DOI:10.2196/30066.

26 Constantinescu EC, Udriștoiu AL, Udriștoiu Ștefan C, *et al.* Transfer learning with pre-trained deep convolutional neural networks for the automatic assessment of liver steatosis in ultrasound images. *Med Ultrason* 2021; **23**: 135–9.

27 Rhyou SY, Yoo JC. Cascaded deep learning neural network for automated liver steatosis diagnosis using ultrasound images. *Sensors* 2021; **21**. DOI:10.3390/s21165304.

28 Huang H, Liu Y, Xiong Q, Xing Y, Du H. A fatty liver diseases classification network based on adaptive coordination attention with label smoothing. *Biomed Signal Process Control* 2023; **86**. DOI:10.1016/j.bspc.2023.105267.

29 Zhu H, Liu Y, Gao X, Zhang L. Combined CNN and Pixel Feature Image for Fatty Liver Ultrasound Image Classification. *Comput Math Methods Med* 2022; **2022**. DOI:10.1155/2022/9385734.

30 Hamaguchi M, Kojima T, Itoh Y, *et al.* The severity of ultrasonographic findings in nonalcoholic fatty liver disease reflects the metabolic syndrome and visceral fat accumulation. *American Journal of Gastroenterology* 2007; **102**: 2708–15.

31 The area under the ROC curve may be a biased performance measure for meta-analysis of diagnostic accuracy studies. A simulation study | Cochrane Colloquium Abstracts. https://abstracts.cochrane.org/2017-cape-town-global-evidence-summit/area-under-roc-curve-may-be-biased-performance-measure-meta (accessed Jan 19, 2025).
